# Nitrogen Balance on Ni–N–C Promotor for High‐Energy Lithium‐Sulfur Pouch Cells

**DOI:** 10.1002/advs.202204027

**Published:** 2022-10-10

**Authors:** Xuan Cao, Menglei Wang, Yuanli Li, Le Chen, Lixian Song, Wenlong Cai, Wei Zhang, Yingze Song

**Affiliations:** ^1^ State Key Laboratory of Environment‐Friendly Energy Materials Tianfu Institute of Research and Innovation School of Materials and Chemistry Southwest University of Science and Technology Mianyang Sichuan 621010 P. R. China; ^2^ College of Energy Soochow Institute for Energy and Materials Innovation Key Laboratory of Advanced Carbon Materials and Wearable Energy Technologies of Jiangsu Province Soochow University Suzhou Jiangsu 215006 P. R. China; ^3^ Department of Advanced Energy Materials College of Materials Science and Engineering Sichuan University Chengdu Sichuan 610064 P. R. China; ^4^ Chongqing Institute of Green and Intelligent Technology Chinese Academy of Sciences Chongqing 400714 P. R. China

**Keywords:** catalytic activity, high energy, lithium‐sulfur pouch cells, Ni–N–C promotor, nitrogen balance

## Abstract

The viability of lithium‐sulfur (Li–S) batteries toward real implementation directly correlates with unlocking lithium polysulfide (LiPS) evolution reactions. Along this line, designing promotors with the function of synchronously relieving LiPS shuttle and promoting sulfur conversion is critical. Herein, the nitrogen evolution on hierarchical and atomistic Ni–N–C electrocatalyst, mainly pertaining to the essential subtraction, reservation and coordination of nitrogen atoms, is manipulated to attain favorable Li–S pouch cell performances. Such rational evolution behavior realizes the “nitrogen balance” in simultaneously regulating the Ni–N coordination environment, Ni single atom loading, abundant vacancy defects, active nitrogen and electron conductivity, and maximizing the electrocatalytic activity elevation of Ni–N–C system. With such merit, the cathode harvests favorable performances in a soft‐packaged pouch cell prototype even under high sulfur mass loading and lean electrolyte usage. A specific energy density up to 405.1 Wh kg^−1^ is harvested by the 0.5‐Ah‐level pouch cell.

## Graphical abstract

The versatile “nitrogen balance” strategy realizes the simultaneously regulation of the Ni—N coordination environment, Ni single atom loading, abundant vacancy defects, active N atoms and electron conductivity, maximizing the electrocatalytic activity elevation of Ni–N–C system, which boosts the electrochemical performance of lithium‐sulfur pouch cells.

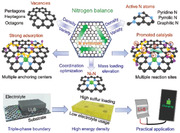

## 1 Introduction

Lithium–sulfur (Li–S) battery serves as an attractive alternative for the current commercial lithium‐ion battery on account of its remarkable merits in theoretical capacity and energy density. Nevertheless, there is still a long way to go for the real implementation of Li–S battery owing to the notorious lithium polysulfide (LiPS) shuttle phenomenon and the retarded sulfur conversion reaction kinetics.^[^ ^1^ ^]^ Of particular note, these issues become more fatal in a working soft‐packaged pouch Li–S cell system, which causes the low capacity and inferior cycling stability, thus giving rise to a distinct energy density difference with the theoretical value.^[^ ^2^ ^]^

The working principle of Li–S battery is the multistep phase transitions of sulfur species along with electron and Li^+^‐ion migration.^[^ ^3^ ^]^ The core of electrocatalysis for Li–S chemistry lies in accelerating the formation and decomposition of Li_2_S in discharge and charge procedures.^[^ ^4^ ^]^ Along this line, confine,^[^ ^5^ ^]^ defect,^[^ ^6^ ^]^ and size engineering^[^ ^7^ ^]^ have been proposed as useful strategies to elevate the activity of electrocatalysts to date, noting that the nitrogen atom usually can endow the promotors with remarkable abilities in controlling the behaviors of LiPSs and Li^+^‐ions. Recently, the combination of nitrogen design with confine,^[^ ^8^ ^]^ defect,^[^ ^9^ ^]^ interface,^[^ ^10^ ^]^ and size engineering^[^ ^11^ ^]^ have been in the spotlight to attain highly active promotors. Our recent work reported the nepenthes‐like and the cubic cage‐shaped N‐doped graphene by the scalable template‐confined synthesis routes for Li−S batteries.^[^ ^8^ ^]^ Such conformal growth of graphene could well preserve the morphologies and architectures of the selected templates, which was conducive to immobilize the LiPSs physically. More importantly, the active nitrogen atoms involving pyridine, pyrrolic, and graphitic N homogeneously on the graphene architecture realized the chemical conversion of LiPSs.

The vacancy defects induced by the nitrogen atom removal also have been substantiated as the active centers for Li−S chemistry.^[^ ^12^ ^]^ We even disclosed that this tunable subtraction of nitrogen atoms under various temperatures can lead to different defect levels, which is beneficial to the activity optimization of carbon promotor for sulfur redox reaction.^[^ ^13^ ^]^ Downsizing the size of catalysts serves as another effective strategy toward activity improvement. Along this line, single atom catalysts (SACs) have emerged as a new frontier owing to their nearly 100% atom utilization efficiency. Of particular note, the nitrogen‐enabled coordination environment boosts the electrocatalytic functions of SACs in Li–S batteries and other electrocatalysis realms.^[^ ^14^ ^]^ Beyond that, the pioneering investigations by Zhang's group proposed that the “lithium bond chemistry” based on the interaction between these active nitrogen atoms and Li^+^‐ions not only affected the sulfur evolution but also was favorable for manipulating the working state of lithium anode.^[^ ^15^ ^]^ Despite of fruitful achievements, the rational regulation of nitrogen evolution behavior targeted active catalyst design and the related mechanism deciphering are urgently desired in the Li−S realm.

In this article, we propose an all‐in‐one “nitrogen balance” strategy for constructing a highly active Ni–N–C system by regulating the local nitrogen evolution behavior targeting high‐energy Li–S pouch cells. The local nitrogen balance leads to the simultaneous optimization of Ni coordination configuration, Ni atom loading, N vacancy defect, active N and electron conductivity, resulting in remarkable active Ni–N–C system which is distinctly different from the traditional Ni SACs. Benefiting from the maximized electrocatalytic activity, the Ni–N–C system effectively triggers the nucleation and decomposition reaction of Li_2_S during the discharge and charge procedure, respectively. Therefore, the electrochemical performances of Li–S pouch cells are remarkably boosted even at a high sulfur loading of 8.3 mg cm^−2^ and relatively low electrolyte dosage of 3.0 µL g^−1^_S_ as well as the folding state. Particularly, a specific energy density of 405.1 Wh kg^−1^ is attained by the Ni–N–C‐enabled 0.5‐Ah‐level pouch cell.

## 2 Results and Discussion

### 2.1 Illustrating a “Nitrogen Balance” Concept for Designing a Highly Active Ni–N–C System

**Figure 1:**
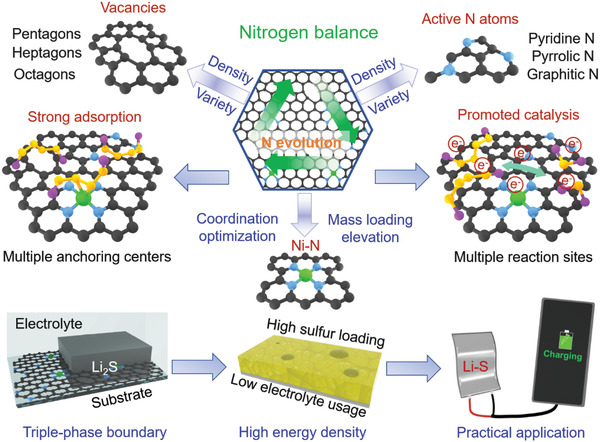
Schematic illustration of all‐in‐one “nitrogen balance” for maximizing the electrocatalytic activity of Ni–N–C system.

**Figure** **1** schematically illustrates the “nitrogen balance” concept targeted the Ni–N–C system with maximized electrochemical activity for Li–S chemistry. The active centers involving the Ni–N coordination, nitrogen vacancy defect and active nitrogen atoms are strongly correlated with the nitrogen atoms, which implies that the essential subtraction and reservation of nitrogen atoms by varying the annealing temperature can lead to the density manipulation of such active centers in carbon architecture. In detail, the essential subtraction of nitrogen atoms results in the density increase of nitrogen vacancies. And the essential reservation of nitrogen atoms lead to the density increase of coordinated Ni–N centers as well as the intrinsic active nitrogen atoms. Therefore, the balanced regulation of nitrogen atoms by controlling the annealing temperature can realize the density of active centers and thus maximize the activity of Ni–N–C system. In the discharge–charge procedures, the “nitrogen balance” strategy‐enabled Ni–N–C system can guide the sulfur species evolution by the chemical adsorption and electrochemical catalysis effect, noting that such a “nitrogen balance” rationale fulfills the comprehensive regulation of active site number and variety, which is favorable for promoting the electrochemical reaction occurring on the triple‐phase boundary generated by LiPS, electrolyte, and Ni–N–C system. Therefore, favorable energy density can be expected to be gained by the Li–S pouch cell under the real scenarios of high sulfur mass loading as well as low electrolyte usage.

**Figure 2:**
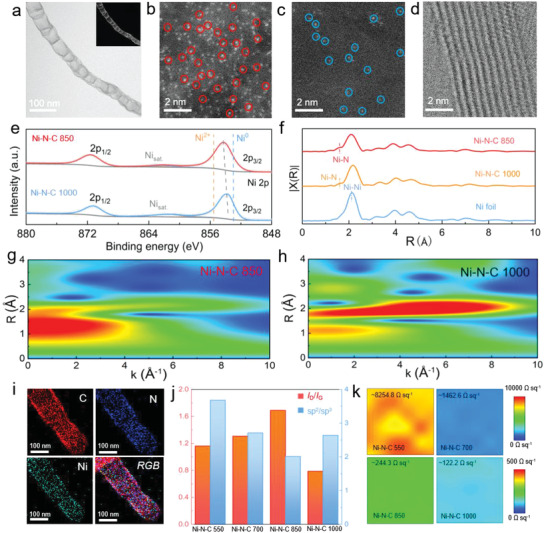
Structural characterizations of the Ni–N–C electrocatalyst. a) TEM images of the Ni–N–C 850 sample. b) HAADF‐TEM images of the Ni single atoms (marked by the red circles) inside the Ni–N–C 850 and c) Ni–N–C 1000 (marked by the blue circles). d) TEM image of the tubular structure inside Ni–N–C 850. e) High‐resolution XPS Ni 2p spectra of Ni–N–C 850 and Ni–N–C 1000, respectively. f) Ni K‐edge EXAFS of Ni–N–C 850, Ni–N–C 1000, and Ni foil. Wavelet‐transformed k^3^‐weighted EXAFS spectra of g) Ni–N–C 850 and h) Ni–N–C 1000. i) The element maps of Ni–N–C 850 sample. j) The *I*_D_/*I*_G_ and sp^2^/sp^3^ values of various Ni–N–C samples according to the Raman and XPS spectra, respectively. k) Sheet resistance maps of various Ni–N–C samples.

Ni(HNCN)_2_ precursor is simultaneously selected as the Ni, N, and C sources. The formed Ni seeds promote the carbon deposition to grow carbon nitride (CN) nanotube based on a vapor–liquid–solid reaction mechanism.^[^ ^16^ ^]^ Meanwhile, the Ni atoms tend to diffuse and thus occupy the unsaturated carbon vacancies driven by the strong Lewis acid–base Ni–N interaction. The programmable optimization among Ni–N coordination, Ni single atom content, doped active nitrogen atoms, vacancy density, and conductivity is conducted by precisely tuning the heating temperature from 550 to 1000 °C (Figures S1 and S2, Supporting Information). The obtained samples at 550, 700, 850 and 1000 °C after acid‐etching procedure are denoted as Ni–N–C 550, Ni–N–C 700, Ni–N–C 850 and Ni–N–C 1000, respectively. It can be clearly observed from the transmission electron microscopy (TEM) image of Ni–N–C 850 that the propelled carbon deposition by Ni catalyst leads to the formation of a unique tubular structure that acts as the superior substrate for Ni atom loading (**Figure** **2**a and Figure S3, Supporting Information). In addition, the observed hollow interior along the length is conduced to the electrolyte permeation and Li^+^‐ion migration, which is of utmost importance to propelling the efficiency of electrochemical reactions and thus enhancing the battery performance. As witnessed by the high‐angle annular dark‐field TEM (HAADF‐TEM) image of Ni–N–C 850, the isolated Ni single atoms are dominantly distributed into the CN substrate, guaranteeing high Ni atom utilization in the electrocatalytic sulfur redox reaction (Figure 2b). When the heating temperature is elevated to 1000 ^°^C, the mass loading of Ni single atoms is significantly reduced (Figure 2c). This is probably due to the fact that the removal of coordinated N atoms and the reconstruction of carbon atoms resulting from the high temperature leads to the detachment of Ni single atoms. The high‐resolution TEM inspection in Figure 2d shows the ten‐layered Ni–N–C 850 tubular structure.

The X‐ray diffraction patterns of samples are exhibited in Figure S4, Supporting Information. The X‐ray photoelectron spectroscopy (XPS) Ni 2p signals for all the Ni–N–C samples are displayed in Figure 2e, and Figures S5 and S6, Supporting Information. As displayed in Figure 2e, the Ni 2p_3/2_ peak switches toward lower low binding energy manifesting that the Ni–N binding interaction becomes weak when the reaction temperature varies from 850 to 1000 °C. The Fourier transform analysis of extended X‐ray absorption fine structure (FT‐XAFS) was conducted to elucidate the coordination environment as exhibited in Figure 2f and Figure S7, Supporting Information. The Fourier transform analysis of extended X‐ray absorption fine structure (FT‐XAFS) spectra of both Ni–N–C 850 and Ni–N–C 1000 present a new Ni–N coordination signal at 1.42 Å which is quite different from the bulge at 1.35 Å in the spectrum of the bare Ni foil. It's noteworthy that a strong Ni–Ni signal (2.15 Å) further depicts the existence of residual Ni particle catalyst within the inner of carbon tubular structure. Despite the many attempts that have been presented such as diluted HCl etching, it's very challenging for the current steps to remove the Ni particle catalyst completely. From a different perspective, the residual Ni particles may represent other active sites of the whole electrocatalytic system. The further wavelet transforms contour plots in Figure 2g,h confirm the Ni–N coordination existing in the Ni–N–C 850 and Ni–N–C 1000 samples (Figure S8, Supporting Information). In addition, the Ni—Ni bonding decreases when the heat treat temperature alters from 850 to 1000 °C, in accordance with the results of HAADF‐TEM images. The scanning TEM and elemental map image of Ni–N–C 850 display the homogeneous spatial distribution of Ni and N onto the carbon architecture (Figure 2i). Different from the Ni–N–C 850, the Ni–N–C 1000 sample shows a relatively small amount of Ni and N elements according to the observed map result (Figures S9 and S10, Supporting Information). The above evidences substantiate the successful manipulation of Ni–N–C system toward high Ni single atom loading. In light of a small amount of Ni particles existing in the material, we combined the inductively coupled plasma‐optical emission spectroscopy (ICP‐OES) and XPS characterizations to detect the content of Ni single atoms. According to the XPS andICP‐OES measurement results, the Ni single atoms of Ni–N–C 550, Ni–N–C 700, Ni–N–C 850 and Ni–N–C 1000 samples are 9.8 wt%, 12.3 wt%, 9.3 wt% and 3.1 wt%, respectively.

The nitrogen vacancies represent the second active centers for Li–S redox reactions, respectively. According to the current literatures, such high temperature‐triggered nitrogen vacancy defects involve pentagons, heptagon and octagons.^[^ ^9^ ^]^ Along this line, the defect density of Ni–N–C system was tuned by controlling the temperature parameter. And the exhaustive characterizations of defect density were fulfilled by comparing the values of *I*_D_/*I*_G_ and sp^2^/sp^3^ based on Raman and XPS spectra, respectively. As seen from Figure S11, Supporting Information, the values of *I*_D_/*I*_G_ for Ni–N–C 550, Ni–N–C 700, Ni–N–C 850 and Ni–N–C 1000 samples are 1.16, 1.31, 1.69 and 1.01, respectively. The sp^2^/sp^3^ values of Ni–N–C 550, Ni–N–C 700, Ni–N–C 850 and Ni–N–C 1000 samples are respectively 3.68, 2.71, 2.01 and 2.64 (Figure S12, Supporting Information). Further, the histogram of defect density stemming from the statistic ratio values of *I*_D_/*I*_G_ and sp^2^/sp^3^ are also exhibited in Figure 2j, indicative of the highest defect density of Ni–N–C 850 sample among the four samples. It can be observed that the defect density is higher due to the increase of nitrogen vacancies, however, becoming lower on account of a new ordered atom arrangement. The same variation trend of defect density can be verified by the previous investigation.^[^ ^13^ ^]^ The partially retained isolated nitrogen atoms in the carbon architecture act as the third active center for Li–S chemistry. The three isolated nitrogen configurations involving of pyridinic, pyrrolic, and graphitic nitrogen can be attained by Ni–N–C 850 and Ni–N–C 1000 samples (Figures S13 and S14, Supporting Information). With the increase of temperature, the nitrogen content varies from 38.1 to 3.2 atom percentage, manifesting the tunable window of defect density and Ni single atom loading. Impressively, the Ni–N–C 850 sample still maintains the overwhelming nitrogen content of 9.2 atom percentage in contrast to the reported doped levels. The conductivity change resulting from temperature operation also plays a critical role in the activity regulation of Ni–N–C for Li–S chemistry. The electron conductivities of samples were explored accordingly. As displayed in Figure 2k and Figure S15, Supporting Information, the average sheet resistance values of 8254.8, 1462.6, 244.3 and 122.2 Ω sq^−1^ are achieved by the Ni–N–C 550, Ni–N–C 700, Ni–N–C 850 and Ni–N–C 1000 samples, respectively, indicating the superior electron conductive properties of latter two samples. Ni–N–C 850 and Ni–N–C 1000 samples show the Brunauer–Emmett–Teller (BET) surface area of 170.6 and 186.6 m^2^ g^−1^, respectively, the values of which are much larger than that of Ni–N–C 550 and Ni–N–C 700 samples (0.8 and 133.6 m^2^ g^−1^, respectively), suggesting their abundant active interfaces for sulfur conversion reactions (Figures S16 and S17, Supporting Information). These results demonstrate 850 °C as the preferred nitrogen evolution temperature for fulfilling the delicate balance of triple active centers, loading content, conductivity and surface area, probably leading to the maximized electrocatalytic activity of Ni–N–C system.

### 2.2 Evaluating the Catalytic Activity of Ni–N–C System

**Figure 3:**
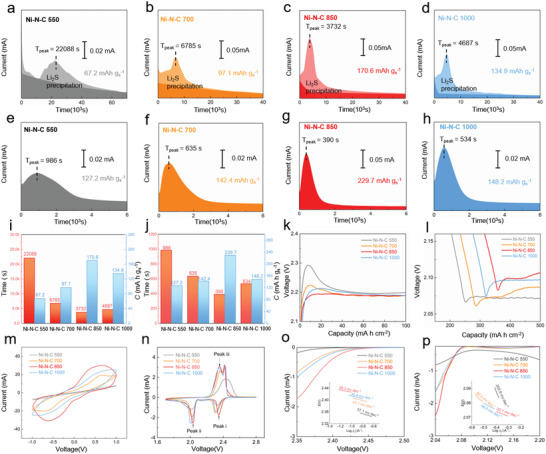
The electrocatalytic activity analysis of the various Ni–N–C electrocatalysts for sulfur conversion reactions. Li_2_S nucleation curves on a) Ni–N–C 550, b) Ni–N–C 700, c) Ni–N–C 850, and d) Ni–N–C 1000 at 2.05 V. Li_2_S decomposition curves on e) Ni–N–C 550, f) Ni–N–C 700, g) Ni–N–C 850, and h) Ni–N–C 1000 at 2.35 V. i) Li_2_S nucleation and j) Li_2_S decomposition quantity and time statistics of various Ni–N–C electrocatalysts. k) Charge and l) discharge curves of various S/Ni–N–C substrates cathodes at 0.1 C. m) CV profiles of various Ni–N–C samples in symmetric cells. n) CV curves of the S/Ni–N–C cathodes in a potential window of 1.7–2.8 V at a scan of 0.05 mV s^−1^. (o) and (p) Tafel plots for the LiPS conversion reactions.

The electrocatalytic activity evaluation of Ni–N–C system was performed by a series of systematic electrochemical characterizations. The Ni–N–C loaded on carbon paper (CP) immersing in electrolyte display a number of LiPS‐adsorptive and electron‐conductive interfaces to accelerate Li_2_S nucleation and dissociation in the potentiostatic discharge and charge curves, respectively. As displayed in **Figure** **3**a–d, the quantity of Li_2_S deposits on CP/Ni–N–C 850 is calculated as 170.6 mAh g^−1^_S_, the value of which is larger than that of CP/Ni–N–C 550 (67.2 mAh g^−1^_S_), CP/Ni–N–C 700 (97.1 mAh g^−1^_S_), and CP/Ni–N–C 1000 (134.9 mAh g^−1^_S_). As a stark contrast to the other three samples, CP/Ni–N–C 850 shows a shorter nucleation time, further corroborating that the CP/Ni–N–C 850 serves as favorable nucleation centers to guide the homogeneous precipitation of Li_2_S. The CP/Ni–N–C 850 surface realizes the Li_2_S decomposition quantity of 229.7 mAh g^−1^_S_, the value of which is much higher compared with that of CP/Ni–N–C 550 (127.2 mAh g^−1^_S_), CP/Ni–N–C 700 142.4 mAh g^−1^_S_), and CP/Ni–N–C 1000 (148.2 mAh g^−1^_S_) (Figure 3e–h). Further, the shortest Li_2_S dissociation time among all the samples also proves the guiding effect of Ni–N–C 850 on the decomposition reaction. The histogram of the capacity and time with respect to the Li_2_S nucleation and dissociation is also exhibited in Figure 3i,j. Such a reversible conversion of Li_2_S illustrates that the “nitrogen balance” strategy is feasible for elevating the electrocatalytic activity of Ni–N–C system. Based on the partial curves of discharge and charge plateau, the Ni–N–C 850 endows the cathode with the lower overpotentials compared with the other three samples (Figure 3k,l), implying its rational electrocatalytic activity design. The cyclic voltammograms (CV) profiles of symmetric cells with two identical CP/Ni–N–C electrodes serving as the working and counter electrodes were recorded to compare their redox current under a polarization of 1.0 V. As shown in Figure 3m, the increased redox current can be harvested by the CP/Ni–N–C 850 in comparison with other three electrodes, confirming the remarkable electrocatalytic ability for sulfur conversion reactions. For the CV curves of the samples in Figure 3n, both the two reduction peaks (i and ii) of S/Ni–N–C cathode shift toward the lower potential due to the rational “nitrogen balance” strategy. The onset potentials of S/Ni–N–C 850 cathode extracted from the CV curves for the discharge processes are remarkably higher than those of other three cathodes (Figure S18, Supporting Information). In addition, the S/Ni–N–C 850 cathode attains the lowest onset potential for the charge procedure among the four cathodes. The onset potential evidence further substantiates the superior electrocatalytic ability of S/Ni–N–C 850 for the sulfur reduction and oxidation reactions.

The Tafel slopes were attained from the linear CV curves to identify the electrocatalytic impact on the charge‐transfer kinetics. In the first conversion step of S_8_ to Li_2_S_4_, the Tafel slope value of S/Ni–N–C 850 is 39.3 mV dec^−1^, which is lower than those of S/Ni–N–C 550 (57.1 mV dec^−1^), S/Ni–N–C 700 (47.1 mV dec^−1^) and S/Ni–N–C 1000 (45.4 mV dec^−1^) (Figure 3o). In the second conversion step of Li_2_S_4_ to Li_2_S_2_/Li_2_S, the lower Tafel slope value of 33.1 mV dec^−1^ is obtained by the S/Ni–N–C 850 in sharp contrast with that of S/Ni–N–C 550 (202.8 mV dec^−1^), S/Ni–N–C 700 (82.0 mV dec^−1^) and S/Ni–N–C 1000 (46.0 mV dec^−1^) (Figure 3p). Such lowered Tafel slope values of S/Ni–N–C 850 substantiates the propelled LiPS conversion reaction kinetics by S/Ni–N–C 850. Electrochemical impedance spectroscopy (EIS) profiles in Figure S19, Supporting Information, reveal that both the fresh Ni–N–C 850 and Ni–N–C 1000 based batteries present lower charge transfer resistance values of 34.2 and 29.4 Ω, respectively, compared with that of other two batteries. To investigate the contribution of residual Ni particles to sulfur evolution, the visual Li_2_S_4_ adsorption behavior and CV profiles of S/bare Ni in symmetric cell were carried out. The Li_2_S_4_ solution still maintains the yellow color even after the addition of Ni particles (Figure S20, Supporting Information). The CV profiles of S/bare Ni cathode in symmetric cell show a slightly obvious redox current than S/Ni–N–C 550 cathode implying the weak ability for accelerating sulfur redox reaction kinetics (Figure S21, Supporting Information). Quite different from the Ni single atoms, the residual Ni particles only show the weak contribution to sulfur evolution based on the above experimental results.

**Figure 4:**
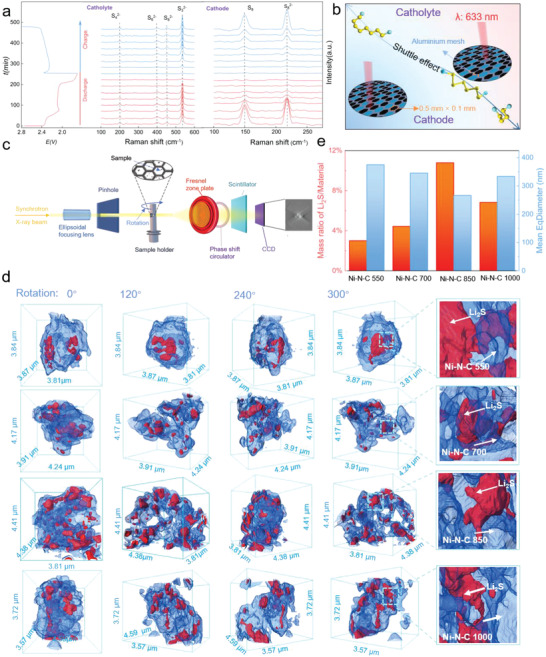
The electrocatalytic mechanism of Ni–N–C samples for Li–S chemistry. a) Operando Raman spectra of Ni–N–C cathode and catholyte on separator upon discharge and charge at 0.2 C. b) Schematic diagram of operando Raman device showing the cathode and catholyte regions. c) Schematic diagram of synchrotron X‐ray 3D nano‐CT facility for electrocatalytic activity evaluation. d) Synchrotron X‐ray 3D nano‐CT images with respect to Li_2_S deposits on Ni–N–C substrates under various rotation angles. e) The statistic volume ratio of Li_2_S and Ni–N–C, mean equivalent diameter of Li_2_S deposits based on the synchrotron X‐ray 3D nano‐CT analysis.

To deeply probe the regulation effect of S/Ni–N–C 850 on sulfur evolution behaviors on line, the combined advanced characterizations of operando Raman spectra and synchrotron 3D nano‐computed tomography (X‐ray 3D nano‐CT) were conducted. Operando Raman spectra act as a preferred toolset for the real‐time observations of soluble LiPS evolution behaviors.^[^ ^17^ ^]^ In consideration of the LiPS shuttle pathways of cathode to anode side, operando Raman spectra based on the observations of catholyte and cathode region were collected during the whole discharge and charge procedures. The catholyte region refers to the catholyte located on separator. The cathodes were obtained by producing the disconnected slurry on Al mesh with a pore size of 0.5 mm × 0.1 mm. For the catholyte region, the Raman signals of S_3_^2−^ (≈535 cm^−1^), S_4_^2−^ (≈202 cm^−1^), S_6_^2−^ (≈397 cm^−1^) and S_8_^2−^ (≈454 cm^−1^) first gradually din the discharge procedure and subsequently increase in the charge process, are clearly witnessed in **Figure** **4**a. The cathode region shows the characteristic peaks of S_8_ (≈150 cm^−1^) and S_8_^2−^ (≈217 cm^−1^) along with the same variation trend as the catholyte region. According to the previous investigations by others and us, bare sulfur cathode and the corresponding catholyte regions present the signals of sulfur species throughout the whole cycling process.^[^ ^18^ ^]^ The Raman signal difference suggests the remarkable regulation effect of S/Ni–N–C 850 on sulfur evolution behaviors (Figure 4b). Synchrotron X‐ray 3D nano‐CT is emerging as a powerful synchrotron facility for deciphering the working mechanism of electrocatalysts (Figure 4c).^[^ ^19^ ^]^ The 3D visualization of Li_2_S deposits on four Ni–N–C substrates based on various rotation angles in Figure 4d and Figure S22, Supporting Information, represents the true depiction of Li_2_S nucleation and growth reaction including the critical homogeneity, size and proportion information. Compared with the other three Ni–N–C substrates, Li_2_S deposit realizes a smaller size and more homogeneous spatial distribution into the inner and external surface of Ni–N–C 850. The mass proportion and diameter information of Li_2_S on Ni–N–C substrates are statistically displayed in Figure 4e. In detail, the mass proportions of Li_2_S and Ni–N–C 550, Ni–N–C 700, Ni–N–C 850 and Ni–N–C 1000 are 3.0%, 4.4%, 10.8% and 6.8%, respectively. Ni–N–C 850 fulfills Li_2_S precipitation with much lower statistical average diameter of (266.4 nm) in contrast with N–C 550 (374.9 nm), Ni–N–C 700 (345.4 nm) and Ni–N–C 1000 (333.9 nm). The synchrotron X‐ray 3D nano‐CT results corroborate the overwhelming electrocatalytic activity of Ni–N–C 850 on account of the versatile “nitrogen balance” strategy.

### 2.3 Deciphering the Catalytic Mechanism of Ni–N–C System

**Figure 5:**
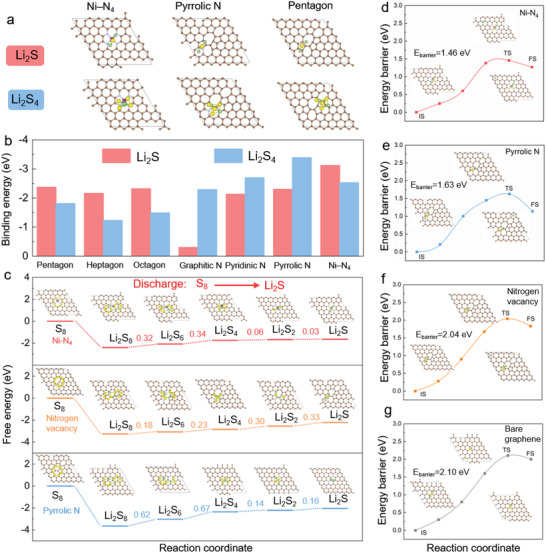
Calculation simulations of sulfur species evolution on various active centers in Ni–N–C. a) Optimized configurations of Li_2_S and Li_2_S_4_ absorption on Ni—N_4_, pyrrolic nitrogen, and pentagon. b) The corresponding adsorption energies of Li_2_S and Li_2_S_4_ on pentagon graphene, heptagon graphene, octagon graphene, graphitic nitrogen, pyridinic nitrogen, pyrrolic nitrogen, and Ni—N_4_ configuration, respectively. c) Gibbs free energy curves for the sulfur species evolution on Ni—N_4_, nitrogen vacancy and pyrrolic nitrogen, respectively, show the corresponding optimized adsorption conformations. Dissociation energy barriers of Li_2_S on d) Ni—N_4_, e) pyrrolic nitrogen, f) nitrogen vacancy, and g) bare graphene, respectively.

To figure out the mechanism for sulfur redox reaction kinetics elevation of Ni–N–C, the first‐principle calculations based on various active centers were carried out. **Figure** **5**a,b and Figure S23, Supporting Information, display the configurations of different active centers involved in the Ni–N–C system. Li_2_S_4_ and Li_2_S species respectively act as the precursor and final product of nucleation reaction. Ni—N_4_ was selected as the representative of coordinated configuration for calculation simulation. Accordingly, it's necessary to calculate their binding energies with different active centers. For the active centers, the binding energies of Li_2_S_4_ by Ni—N_4_, pyrrolic nitrogen, pyridinic nitrogen, graphitic nitrogen, pentagon, heptagon and octagon configuration are 2.53, 3.39, 2.69, 2.29, 1.81, 1.23 and 1.49 eV, respectively. And the Li_2_S binding energies with Ni—N_4_, pyrrolic nitrogen, pyridinic nitrogen, graphitic nitrogen, pentagon, heptagon and octagon configuration are respectively 3.12, 2.30, 2.13, 0.31, 2.37, 2.16 and 2.32 eV. In addition, the LiPS binding energies for pyridinic and graphitic nitrogen configuration are also exhibited. In contrast, the visual Li_2_S_4_ adsorption measurements were also performed. On account of its direct precursor role in Li_2_S nucleation and growth reaction procedure, Li_2_S_4_ solution was used as the LiPS representative for adsorption test. As seen from Figure S24, Supporting Information, the yellow Li_2_S_4_ solution fades completely in 2.0 h after the introduction of Ni–N–C powder, showing a stronger anchoring capability due to the ample active centers.

The stepwise sulfur evolution pathways on the various active centers were probed by attaining optimal configurations and calculating the corresponding Gibbs free energies (Figure 5c and Figure S25, Supporting Information). The Ni—N_4_, pyrrolic nitrogen, pyridinic nitrogen, graphitic nitrogen and nitrogen vacancy configuration achieve the largest positive Gibbs free energy values of 0.34, 0.67, 1.16, 0.81 and 0.33 eV, respectively, representing their own rate‐limiting step in discharge procedure, which demonstrates the thermodynamically favorable sulfur conversion reaction on Ni—N_4_ compared to other active centers. To explore the reaction kinetics in charge process, the energy barriers for Li_2_S decomposition on active centers were calculated. As plotted in Figure 5d–g and Figure S26, Supporting Information, the energy barriers for Li_2_S decomposition on Ni—N_4_, pyrrolic nitrogen and nitrogen vacancy configuration are 1.46, 1.63 and 2.04 eV, in sharp contrast with that of bare graphene configuration (2.10 eV), warranting the fast Li_2_S dissociation reaction kinetics. The rational integration of multiple active centers into carbon tubular structure by the nitrogen evolution strategy results in the maximized electrocatalytic impact of Ni–N–C on the formation and decomposition of Li_2_S in discharge and charge procedures. The Ni–N–C 850 sample fulfills synchronously the LiPS affinity and electrocatalytic ability enhancement on account of the synergy and division of different centers (Figure S27, Supporting Information).

### 2.4 Evaluating the Electrochemical Performance of Pouch Cell

**Figure 6:**
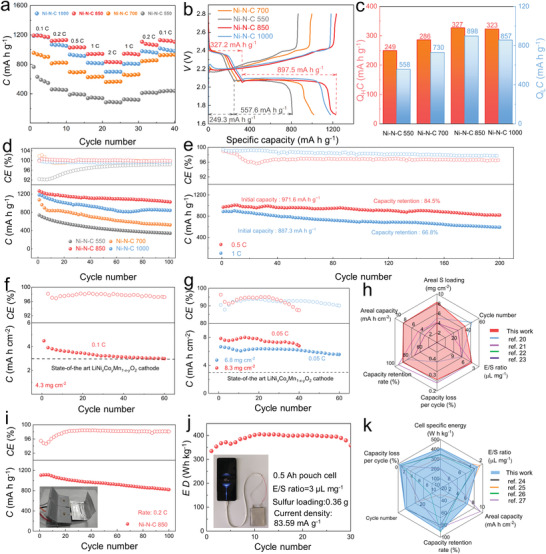
The electrochemical performance of various Ni–N–C as the electrocatalysts for Li–S pouch cells. a) Rate capability of various S/Ni–N–C cathodes. b) Galvanostatic discharge–charge curves of various S/Ni–N–C cathodes at 0.1 C. c) The statistic capacities with respect to the two plateaus of various S/Ni–N–C cathodes. d) Cycling performance of the cathodes at 0.1 C. e) Long‐term cycling performances of S/Ni–N–C 850 cathode at 0.5 and 1 C. Cycling performance of S/Ni–N–C 850 cathode with the sulfur mass loadings of f) 4.3 mg cm^−2^ at 0.1 C, g) 6.8 mg cm^−2^ and 8.3 mg cm^−2^ at 0.05 C. i) Cycling performance of S/Ni–N–C 850 with the folding angle of 90°. j) Capacity retention of 0.36‐g‐sulfur pouch cell with the Ni–N–C 850 as the electrocatalyst, inset: the diagram of charging mobile phone by the two pouch cells. Cell performance comparison of h) high‐sulfur‐load  and k) multi‐layer Li–S pouch cells in this work with the reported pouch cells.

To demonstrate the effect of the highly active Ni–N–C concept on practically operated Li–S pouch cells, we first prepared routine cathodes with a sulfur mass loading of 1.7–2.0 mg cm^−2^. Along this line, the systematic electrochemical performance evaluation of Ni–N–C‐enabled pouch cells were also performed. As shown in **Figure** **6**a, the S/Ni–N–C 850 cathode harvests the capacities of 1192.5, 1122.8, 1027.2, 938.3 and 834.3 mA h g^−1^ at a rate of 0.1, 0.2, 0.5, 1 and 2 C, respectively, the values of which are much higher than those of other samples under the same testing rates, implying its more remarkable rate capability. More impressively, the S/Ni–N–C 850 cathode can still remain the highest capacity recovery of 1129.2 mA h g^−1^ among all the samples including S/Ni–N–C 1000 (1016.2 mA h g^−1^), S/Ni–N–C 700 (923.4 mA h g^−1^), and S/Ni–N–C 550‐optimized pouch cells (431.4 mA h g^−1^) as the rate shifts back to 0.1 C. The related discharge–charge profiles of the cathodes have been illustrated in Figure 6b, noting that the Ni–N–C 850 can even endow the pouch cell with prolonged low‐voltage plateaus at a rate of 0.1 C than the other three promotors, substantiating the superior ability of Ni–N–C 850 model for immobilizing LiPS shuttle and propelling LiPS conversion. The corresponding capacities with regards to the two plateaus in the discharge curves of various Ni–N–C‐based pouch cells are also exhibited in Figure 6c.

As shown in Figure 6d, the S/Ni–N–C 850 cathode harvests a higher initial capacity of 1265.4 mA h g^−1^ and superior retention of 81.3% at 0.1 C after 100 cycles compared with S/Ni–N–C 1000 (1181.2 mA h g^−1^ and 71.3% capacity retention), S/Ni–N–C 700 (1072.3 mA h g^−1^ and 48.7% capacity retention), and S/Ni–N–C 550 (737.5 mA h g^−1^ and 46.1% capacity retention). The severe shuttle effect during the initial cycling period resulting from the inferior electron conductivity, relatively low surface area, and porosity of Ni–N–C 550 leads to a large Coulombic efficiency difference between S/Ni–N–C 550 and other three cathodes. It's noted that the S/Ni–N–C 850 cathode can still remain cycling for 300 cycles, further demonstrating its effective sulfur utilization as well as remarkable cycling stability (Figure S28, Supporting Information). The S/Ni–N–C 850 cathode maintains superior structural integrity than that of the S/Ni–N–C 1000 at 0.1 C after 100 cycles (Figure S29, Supporting Information). According to the HAADF‐TEM image of Ni–N–C 850 after cycling, no agglomeration of Ni single atom can be observed, substantiating the remarked stability of Ni‐SAC (Figure S30, Supporting Information). The high‐resolution XPS Ni 2p and N 1s spectra of Ni–N–C 850 before and after cycling show that there are no obvious peak shifts, which demonstrates the stable atomic coordination of Ni single atoms (Figure S31, Supporting Information).

Due to the remarkably elevated electrocatalytic activity, the S/Ni–N–C 850‐based pouch cell presents a high initial capacity of 971.6 mA h g^−1^ as well as capacity retention of 84.5% after 200 cycles at 0.5 C (Figure 6e). When the rate is increased to 1 C, such a pouch cell still holds an initial capacity of 887.3 mA h g^−1^ and favorable cycling performance. The as obtained superior long‐term stability proves the optimization effect of highly active Ni–N–C on the lifespan of the pouch cells. To further evaluate the potential of the Ni–N–C 850‐based pouch cell toward commercial viability, the sulfur mass loading along with the electrolyte/sulfur ratio (E/S) was controlled. The E/S value for high‐sulfur‐load cathodes is 3.5 µL mg_S_^−1^. In Figure 6f, the S/Ni–N–C 850 cathode with the sulfur mass loading of 4.3 mg cm^−2^ respectively delivers the initial and final capacities of 4.0 and 3.0 mA h cm^−2^ after 60 cycles at 0.1 C, respectively. A remarkable capacity of 6.7 mA h cm^−2^ and a retention of 5.6 mA h cm^−2^ after 60 cycles at 0.05 C are also gained by the S/Ni–N–C 850 cathode with the sulfur mass loading of 6.8 mg cm^−2^. When the sulfur loading is continued to elevate to 8.3 mg cm^−2^, an areal capacity of 7.9 mA h cm^−2^ as well as a retention of 6.8 mA h cm^−2^ at 0.05 C after 40 cycles are attained by the S/Ni–N–C 850 cathode. The cathode thickness for sulfur mass loading of 6.8 and 8.3 mg cm^−2^ is 46 and 79 µm, respectively, according to the SEM observation (Figure S32, Supporting Information). On account of the effective activity manipulation of S/Ni–N–C 850, the pouch cells can hold superior electrochemical performances with the sulfur mass loading varying from 4.3 to 8.3 mg cm^−2^ in contrast to the commercial LiNi*_x_* Co*_y_* Mn_1−_ *_x_*_−_ *_y_* O_2_‐based cathodes.

The Ni–N–C 850‐enabled pouch cell can also realize the power supplies to a light‐emitting diode (LED) device under the different folding times (Figure S33, Supporting Information), noting that the voltage of a pouch cell can be stable when the folding time increases to 200 (Figure S34, Supporting Information). Further, the electrochemical performance of a pouch cell with the folding angle of 90° was also evaluated. As plotted in Figure 6i, the pouch cell still delivers an outstanding initial capacity of 1104.2 mA h g^−1^ and 0.25% per cycle at 0.2 C over 100 cycles. These results further corroborate the feasibility of implementing the “nitrogen balance” strategy on Ni–N–C system. To fulfill the energy density goal of 300 Wh kg^−1^, the E/S value is commonly controlled to 4.0 µL mg^−1^_S_. As such, we fabricated a 0.5A‐h‐level pouch cell with the sulfur mass loading of 0.36 g sulfur and the E/S usage of 3.0 µL mg^−1^_S_ (Figure S35, Supporting Information). Accordingly, the specific energy density of a pouch cell with 0.36 g sulfur loading can reach 405.1 Wh kg^−1^ (Figure 6j and Figure S36, Supporting Information). And the calculated volumetric energy density can be up to 434.0 Wh L^−1^ (Figure S37, Supporting Information). And such a 0.5A‐h‐level pouch cell can hold the stable cycling for 30 cycles (≈94% capacity retention), supporting the effectiveness of our “nitrogen balance” strategy on maximizing the electrocatalytic activity of the Ni–N–C concept. By comparing the recent results originating from the fruitful reports, our highly active Ni–N–C 850 can still endow the pouch cell with overwhelming electrochemical performance under the practical scenario of large sulfur loading (Figure 6h) [20–23] and multi‐layer cathodes (Figure 6k) [24–27]. The multiple active centers involving coordinated Ni single atoms, nitrogen vacancy defects and active nitrogen atoms make full use of their own advantages in atom utilization efficiency, LiPS anchoring ability and electron/Li^+^‐ion transfer capability to maximize the activity of Ni–N–C system for Li–S redox reactions. According to our calculation simulation results, the active centers also present different abilities for chemically adsorbing LiPSs and electrochemically catalyzing sulfur conversion reactions. Based on the theoretical evidence, the N‐coordinated Ni single atoms overwhelm other centers in the Li_2_S adsorption energy, decomposition energy barrier, and rate‐limiting step. Meanwhile, the high atom utilization efficiency and large mass loading boost these functions of Ni single atoms for Li–S redox reactions. As a consequence, Ni single atoms make the outstanding contribution to the overall activity elevation of Ni–N–C electrocatalyst. As such, the multiple active centers exhibit the synergy and division effect on promoting the Li–S redox reaction kinetics and thus enhancing the battery performance. However, for practically viable pouch cells, the continued elevation of energy density as well as the effective extension of serving lifespan are desired to be tackled from further endeavors from both theoretical and experimental implementations.

## 3 Conclusion

In conclusion, we have successfully conducted a “nitrogen balance” strategy on Ni–N–C system to maximize its electrochemical activity for Li–S chemistry. Such a well‐designed strategy lies in the comprehensive regulation Ni—N coordination environment, Ni single atom loading, abundant vacancy defect density, active nitrogen as well as electron conductivity, benefiting to mitigate the shuttle effect, propelling the Li_2_S nucleation and decomposition reaction kinetics simultaneously. Furthermore, the electrocatalytic activity contrast of the as designed active centers according to the theoretical simulations deciphers the working mechanism of Ni–N–C concept. Benefiting from the maximized electrocatalytic activity of Ni–N–C promotor, the Li–S pouch cells harvest favorable discharge capacity and cycling stability even under the condition of high sulfur loading and 90° folding angle. Impressively, a 0.5A‐h‐level Li–S pouch cell with a remarkable energy density of 405.1 Wh kg^−1^ has also been fulfilled successfully. This work provides an effective strategy of electrocatalyst activity elevation for actually available LSBs, which alleviates the gap between the practical energy of batteries and future power demand.

## Conflict of Interest

The authors declare no conflict of interest.

## Supporting information

Supporting Information

## Data Availability

The data that support the findings of this study are available from the corresponding author upon reasonable request.

## References

[1] a) Y. Chen , T. Wang , H. Tian , D. Su , Q. Zhang , G. Wang , Adv. Mater. 2021, 33, 2003666; 10.1002/adma.202003666 34096100

[2] a) G. Zhou , H. Chen , Y. Cui , Nat. Energy 2022, 7, 312;

[3] a) K. Zou , T. Zhou , Y. Chen , X. Xiong , W. Jing , X. Dai , M. Shi , N. Li , J. Sun , S. Zhang , C. Zhang , Y. Liu , Z. Guo , Adv. Energy Mater. 2022, 12, 2103981;

[4] a) Z. Han , R. Gao , Y. Jia , M. Zhang , Z. Lao , B. Chen , Q. Zhang , C. Li , W. Lv , G. Zhou , Mater. Today 2022, 57, 84;

[5] Y. Song , Z. Sun , Z. Fan , W. Cai , Y. Shao , G. Sheng , M. Wang , L. Song , Z. Liu , Q. Zhang , J. Sun , Nano Energy 2020, 70, 104555.

[6] a) W. Hou , P. Feng , X. Guo , Z. Wang , Z. Bai , Y. Bai , G. Wang , K. Sun , Adv. Mater. 2022, 34, 2202222; 10.1002/adma.202202222 35534022

[7] a) J. Lei , X. X. Fan , T. Liu , P. Xu , Q. Hou , K. Li , R. M. Yuan , M. S. Zheng , Q. F. Dong , J. J. Chen , Nat. Commun. 2022, 13, 202; 35017484 10.1038/s41467-021-27866-5 PMC8752791

[8] Q. Li , Y. Song , R. Xu , L. Zhang , J. Gao , Z. Xia , Z. Tian , N. Wei , M. H. Rummeli , X. Zou , J. Sun , Z. Liu , ACS Nano 2018, 12, 10240. 30204407 10.1021/acsnano.8b05246

[9] L. Guan , H. Hu , L. Li , Y. Pan , Y. Zhu , Q. Li , H. Guo , K. Wang , Y. Huang , M. Zhang , Y. Yan , Z. Li , X. Teng , J. Yang , J. Xiao , Y. Zhang , X. Wang , M. Wu , ACS Nano 2020, 14, 6222. 32352746 10.1021/acsnano.0c02294

[10] M. Wang , Y. Song , Z. Sun , Y. Shao , C. Wei , Z. Xia , Z. Tian , Z. Liu , J. Sun , ACS Nano 2019, 13, 13235. 31652045 10.1021/acsnano.9b06267

[11] S. Yu , Y. Sun , L. Song , X. Cao , L. Chen , X. An , X. Liu , W. Cai , T. Yao , Y. Song , W. Zhang , Nano Energy 2021, 89, 106414.

[12] a) M. Wang , Z. Sun , H. Ci , Z. Shi , L. Shen , C. Wei , Y. Ding , X. Yang , J. Sun , Angew. Chem., Int. Ed. 2021, 60, 24558; 10.1002/anie.202109291 34435420

[13] L. Chen , S. Yu , Y. Zhang , Y. Song , L. Song , J. Power Sources 2021, 514, 230607.

[14] a) L. Zhang , D. Liu , Z. Muhammad , F. Wan , W. Xie , Y. Wang , L. Song , Z. Niu , J. Chen , Adv. Mater. 2019, 31, 1903955; 10.1002/adma.201903955 31423664

[15] T. Z. Hou , W. T. Xu , X. Chen , H. J. Peng , J. Q. Huang , Q. Zhang , Angew. Chem., Int. Ed. 2017, 56, 8178. 10.1002/anie.201704324 28520218

[16] C. Zhao , Y. Wang , Z. Li , W. Chen , Q. Xu , D. He , D. Xi , Q. Zhang , T. Yuan , Y. Qu , J. Yang , F. Zhou , Z. Yang , X. Wang , J. Wang , J. Luo , Y. Li , H. Duan , Y. Wu , Y. Li , Joule 2019, 3, 584.

[17] L. Zhang , T. Qian , X. Zhu , Z. Hu , M. Wang , L. Zhang , T. Jiang , J. H. Tian , C. Yan , Chem. Soc. Rev. 2019, 48, 5432. 31647083 10.1039/c9cs00381a

[18] a) M. Wang , Y. Song , N. Wei , Y. Shao , G. Sheng , J. Sun , Chem. Eng. J. 2021, 418, 129407;

[19] J. Zhou , X. Liu , L. Zhu , J. Zhou , Y. Guan , L. Chen , S. Niu , J. Cai , D. Sun , Y. Zhu , J. Du , G. Wang , Y. Qian , Joule 2018, 2, 2681.

[20] L. Luo , J. Li , H. Y. Asl , A. Manthiram , ACS Energy Lett. 2020, 5, 1177.

[21] C. Zhao , G. L. Xu , Z. Yu , L. Zhang , I. Hwang , Y. X. Mo , Y. Ren , L. Cheng , C. J. Sun , Y. Ren , X. Zuo , J. T. Li , S. G. Sun , K. Amine , T. Zhao , Nat. Nanotechnol. 2021, 16, 166. 33230316 10.1038/s41565-020-00797-w

[22] J. He , A. Bhargav , A. Manthiram , Adv. Mater. 2020, 32, 2004741. 10.1002/adma.202004741 32864813

[23] J. Zhang , C. You , J. Wang , H. Xu , C. Zhu , S. Guo , W. Zhang , R. Yang , Y. Xu , Chem. Eng. J. 2019, 368, 340.

[24] C. Qu , Y. Chen , X. Yang , H. Zhang , X. Li , H. Zhang , Nano Energy 2017, 39, 262.

[25] L. Shi , S.‐M. Bak , Z. Shadike , C. Wang , C. Niu , P. Northrup , H. Lee , A. Y. Baranovskiy , C. S. Anderson , J. Qin , S. Feng , X. Ren , D. Liu , X. Q. Yang , F. Gao , D. Lu , J. Xiao , J. Liu , Energy Environ. Sci. 2020, 13, 3620.

[26] C. X. Zhao , X. Y. Li , M. Zhao , Z. X. Chen , Y. W. Song , W. J. Chen , J. N. Liu , B. Wang , X. Q. Zhang , C. M. Chen , B. Q. Li , J. Q. Huang , Q. Zhang , J. Am. Chem. Soc. 2021, 143, 19865. 34761937 10.1021/jacs.1c09107

[27] Z. Fang , Y. Luo , H. Wu , L. Yan , F. Zhao , Q. Li , S. Fan , J. Wang , Carbon 2020, 166, 183.

