# Supplementary material for: Nitrogen Balance on Ni–N–C Promotor for High‐Energy Lithium‐Sulfur Pouch Cells
Source: Adv Sci (Weinh). 2022 Oct 10;9(33):2204027. doi: 10.1002/advs.202204027 (PMC9685457; doi:10.1002/advs.202204027)
Supplement: Supplementary file 1 — Supporting Information [file ADVS-9-2204027-s001.pdf]

ADVANCED SCIENCE
Open Access

Supporting Information

for *Adv. Sci.*, DOI 10.1002/advs.202204027

Nitrogen Balance on Ni–N–C Promotor for High-Energy Lithium-Sulfur Pouch Cells

Xuan Cao, Menglei Wang, Yuanli Li, Le Chen, Lixian Song, Wenlong Cai, Wei Zhang* and Yingze Song**

Supporting Information

Nitrogen balance on Ni–N–C promotor for high-energy lithium-sulfur pouch cells

Xuan Cao, Menglei Wang, Yuanli Li, Le Chen, Lixian Song, Wenlong Cai, Wei Zhang* and Yingze Song**

I. Supporting Text

1 Experimental

1.1 Synthesis of Ni(HNCN)$_2$

In a typical synthesis procedure, 2.38 g $NiCl_2{\cdot}6H_2O$ and 2.00 g H_2NCN were first dissolved into 40 mL deionized water. Then 30 mL $NH_3{\cdot}H_2O$ with the concentration of 6.25 *wt.*% was added dropwise to the above solution under vigorous stirring. After stirring at 40 ^{o}C for 10 h, the as-attained blue precipitates were treated by filtration, washing several times, and finally dried under vacuum at 60 ^{o}C for 12 h.

1.2. Synthesis of Ni–N–C electrocatalysts

Typically, the as-prepared Ni(HNCN)$_2$ powder was transferred into a tubular furnace and maintained at the setting temperature for 90 min under vacuum condition. After cooling to room temperature, the obtained black powders were immersed in the diluted hydrochloric acid solution and stirred mildly for 12 h to remove the Ni residue. Then the as-achieved powders were thoroughly washed with deionized water until pH=7.0. After drying in an oven at 60 °C for 12 h, the Ni–N–C powder was obtained eventually. The pyrolysis temperature was set to 550, 700, 850 and 1000 ^{o}C for preparing Ni–N–C 550, Ni–N–C 700, Ni–N–C 850, Ni–N–C 1000 samples, respectively.

1.3. Preparation of S/Ni–N–C composites

0.3 g sublimed sulfur and 0.1 g Ni–N–C electrocatalyst were mixed vigorously on a twin-shaft mixer for 60 min. The as-derived mixture was sealed into a glass vessel and heated at 155 ^{o}C for 6 h to gain the S/ Ni–N–C composite.

1.4. Materials Characterizations

The morphologies of materials were studied by ZEISS sigma500 scanning electron microscopy (SEM) and transmission electron microscopy (TEM). High-angle annular dark-field scanning transmission electron microscopy (HAADF-STEM) images and elemental maps of materials were recorded on a JEOL Jem 200F TEM/STEM equipped with a spherical

aberration corrector. XRD patterns of samples were obtained on a Nalytical X'Pert Pro diffractometer. Brunauer-Emmett-Teller (BET) surface area and pore size measurements were performed on Micromeritics Autosorb-IQ at 77K. X-ray photoelectron spectroscopy (XPS) was inspected on Thermo Scientific K-Alpha system as reference. The sheet resistance maps of various Ni–N–C sample membranes were characterized with the aid of a four-probe 455 resistance measuring system (Guangzhou 4-probe Tech Co. Ltd, RTS-4). The Ni–N–C membranes were prepared by virtue of vacuum filtration route. 20 mg Ni–N–C powder was dispersed into 50 mL isopropyl alcohol solvent by ultrasonic processing for 10 min. The as-prepared dispersion was filtered on a polytetrafluoroethylene (PTFE) film and subsequently dried at room temperature for 10 h. The inductively coupled plasma-optical emission spectroscopy (ICP-OES) were recorded on PerkinElmer Optima 5300 DV. *Operando* Raman spectra of Li–S batteries were studied by a Horiba LabRAM HR Evolution confocal Raman instrument under the excitation of 633 nm. An LSB-Raman cell (Beijing Scistar Tech. Co. Ltd.) was employed for the *operando* Raman measurement. X-ray absorption fine structure spectra (Ni K-edge) of samples were obtained under ambient conditions at the beamline 1W1B of Beijing Synchrotron Radiation Facility (BSRF), Beijing, China.

1.5. Visual LiPS adsorption tests

Li_2S_4 solution was selected as the representative sulfur species for the visual adsorption test. In detail, Li_2S_4 solution with a concentration of 7.0 mmol L^{-1} was obtained by dissolving the lithium sulfide and sublimed sulfur at a molar ratio of 1:3 in 1, 2-dime-thoxyethane (DME) solvent. Then, the electrocatalyst powder with the identical mass of 50 mg was added into 2 mL Li_2S_4 solution for color observation, respectively. The whole test process was accomplished in an Ar-filled glove box.

1.6. Assembly of symmetric cells

Ni–N–C 550, Ni–N–C 700, Ni–N–C 850, Ni–N–C 1000 powder were loaded on the surface of carbon paper (CP) with a diameter of 13 mm for electrode preparation, respectively. Afterwards, the as-gained electrodes were assembled into symmetric cells. 20 μL Li_2S_6 solution with a concentration of 0.2 mol L^{-1} as the electrolyte and Celgard 2500 membrane as the separator. CV measurements of the symmetrical cells were performed at a scan rate of 50 mV s^{-1} in the potential window of –1.0 to 1.0 V.

1.7. Li_2S nucleation tests

0.2 mol L^{-1} Li_2S_8 solution was prepared by dissolving sublimated sulfur and lithium sulfide into tetraglyme solution at a molar ratio of 7:1. Subsequently, Ni–N–C 550, Ni–N–C 700, Ni–N–C 850, Ni–N–C 1000 powder were loaded on CP disks. All the cathodes were

respectively assembled into LIR 2032-type coin cells by synchronously using lithium foil as the anode, 20 μL of Li_2S_8 solution and 20 μL of LiTFSI (1 mol L^{-1}) solution without Li_2S_8 as the catholyte anolyte, respectively. Li_2S nucleation curves of cells were gathered on a Vertex.One Electrochemical Workstation by first galvanostatically discharging to 2.06 V at 0.112 mA and subsequently potentiostatically discharging at 2.05 V to 10^{-5} A.

1.8. Li_2S dissociation tests

As for the Li_2S decomposition test, the cells were fabricated by following the same procedure with the Li_2S nucleation test. To ensure the complete transformation of LiPSs into Li_2S, the dissociation tests were first performed galvanostatically at a current of 0.112 mA until the potential was below 1.7 V. Subsequently, the cells were potentiostatically charged at 2.35 V to 10^{-5} A.

1.9. Synchrotron X-ray 3D nano-imaging

The synchrotron X-ray 3D nano-CT was studied on the beamline BL07W at the National Synchrotron Radiation Laboratory (NSRL), Hefei, China, (flux: 2 × 1010 Phs s≤ 1, spatial resolution: 30 nm). Firstly, 10.0 mg powder sample after Li_2S nucleation test was uniformly dispersed in ethanol solution. Afterwards, 10.0 μL of the obtained mixer was dropped onto a piece of carbon-free formvar film with 100 mesh, and followed by drying at room temperature. The whole sample preparation procedure was conducted under a pure argon atmosphere. The loaded formvar film was further transferred into the vacuum chamber for imaging. Two-dimensional (2D) tomograms were acquired at a title-angle range of 60◦ to 60◦ with an interval of 1°. The achieved 2D tomograms were reconstructed into 3D tomograms by using Xradia XMReconstructor software to export the reconstructed images. Further 3D visualization and segmentation of the reconstructed images were performed on an Avizo Fire VSG software (Visualization Sciences Group, Bordeaux).

1.10. Theoretical Calculations

Density-functional theory (DFT) calculations in this work were implemented by using the Vienna ab-initio simulation package (VASP), Abinit code [1] and Phonopy code [2]. The projector augmented wave (PAW) method was used to depict electron-ion interactions. Double check by Abinit code [1] were also performed. The Perdew-Burke-Ernzerh version of generalized gradient approximation (GGA-PBE) functional was employed to treat the electronic exchange-correlation energy. Plane-wave basis cutoff energy is set as 500 eV. A supercell of graphene containing 6 × 6 unit cells that contain 72 Carbon atoms was used to construct the adsorption model. The 2-dimensional geometric optimization and self-consistent electronic calculations were performed with 7×7×1 k-mesh to guarantee quality convergence.

Supporting Figures

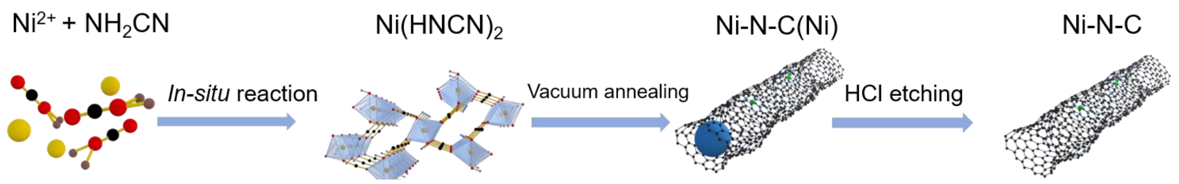

Figure S1. *Schematic diagram of the synthetic process of the Ni–N–C electrocatalyst.*

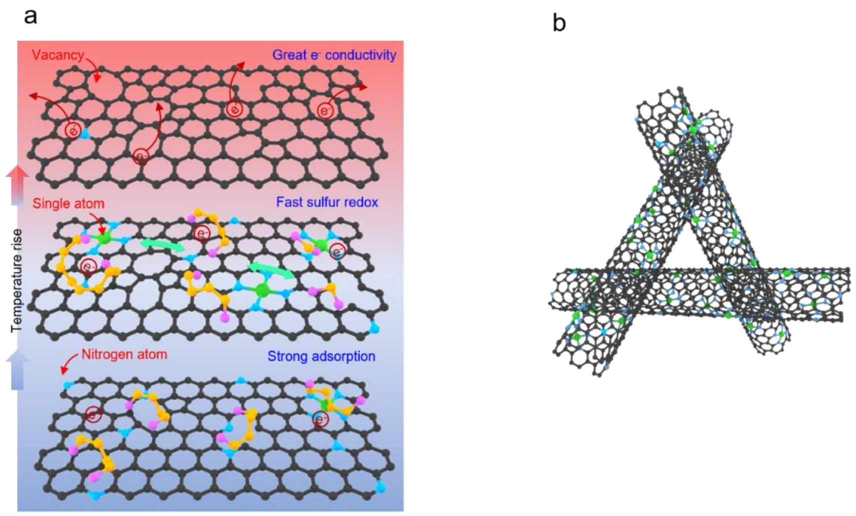

Figure S2. (a) All in one " nitrogen evolution" strategy for Ni–N–C system. (b) Unique tubular structure of Ni–N–C 850.

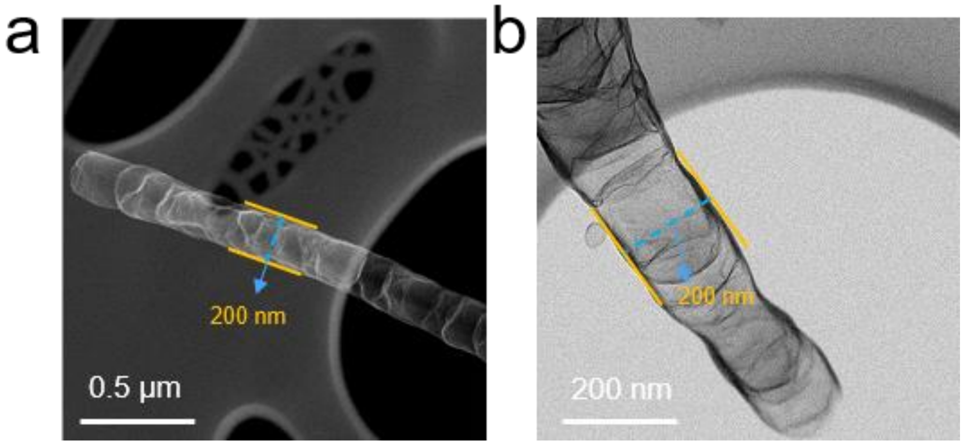

Figure S3. TEM characterizations of Ni–N–C 850.

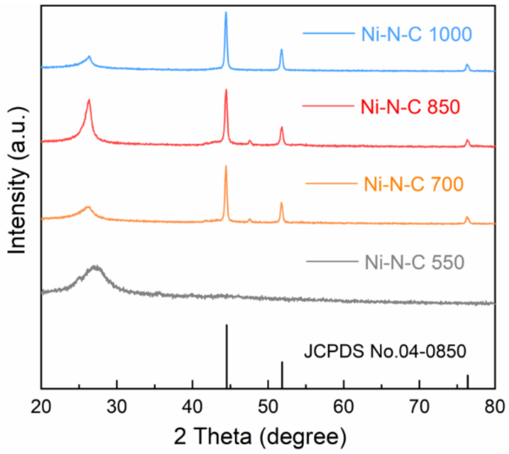

Figure S4. The X-ray diffraction patterns of Ni–N–C samples.

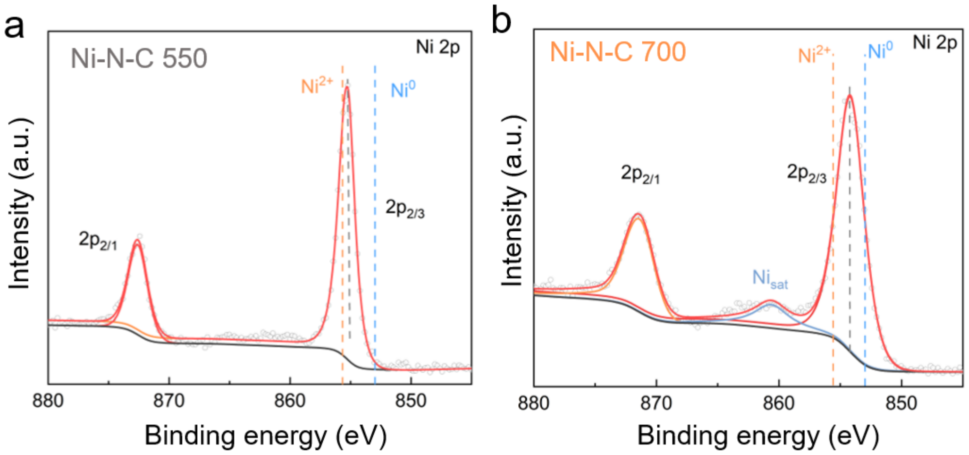

Figure S5. High-resolution XPS Ni 2p spectra of (a) Ni–N–C 550 and (b) Ni–N–C 700 samples.

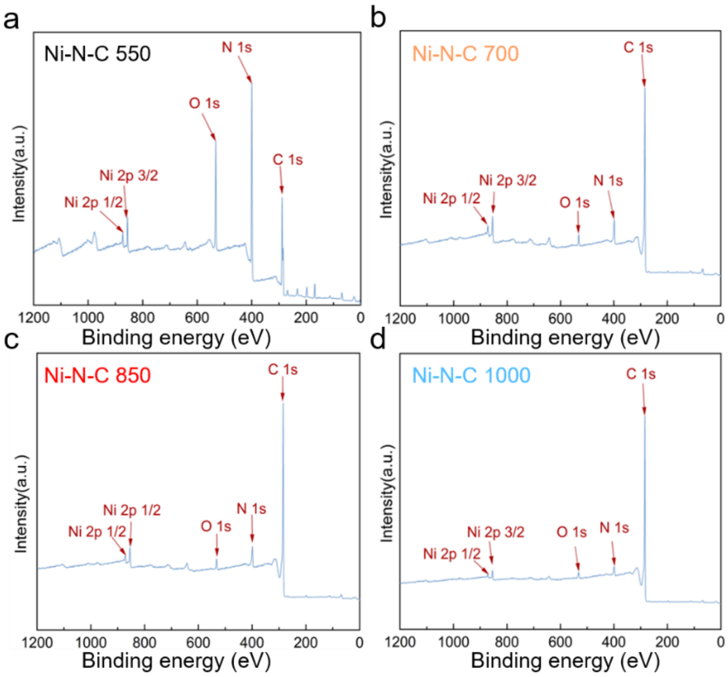

Figure S6. XPS spectra of Ni–N–C (a) Ni–N–C 550; (b) Ni–N–C 700; (c) Ni–N–C 850 and (d) Ni–N–C 1000 samples.

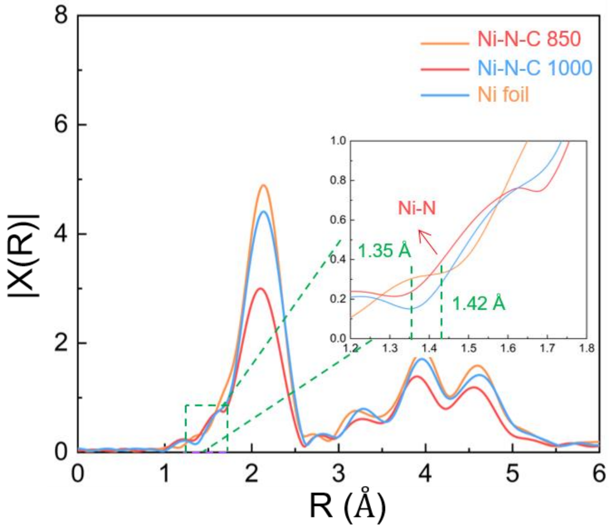

Figure S7. Ni K-edge EXAFS of Ni–N–C 850, Ni–N–C 1000 and Ni foil.

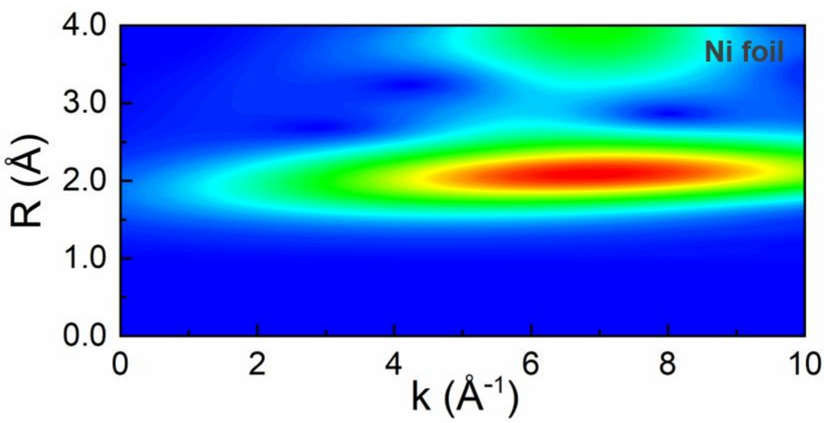

Figure S8. The wavelet transforming plot of Ni foil.

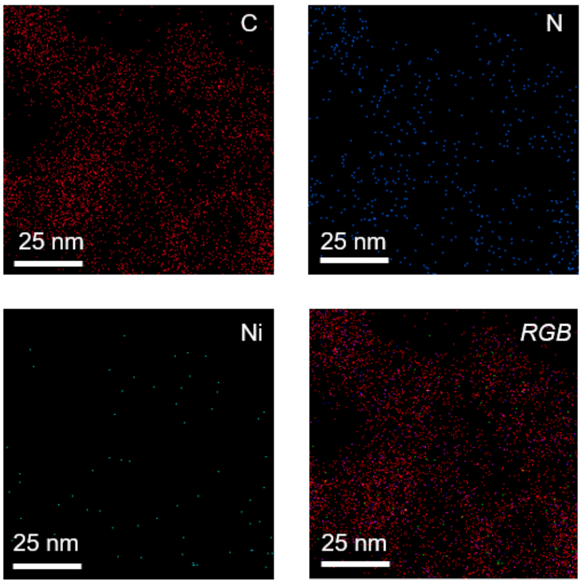

Figure S9. High-resolution TEM image of Ni–N–C 1000 sample.

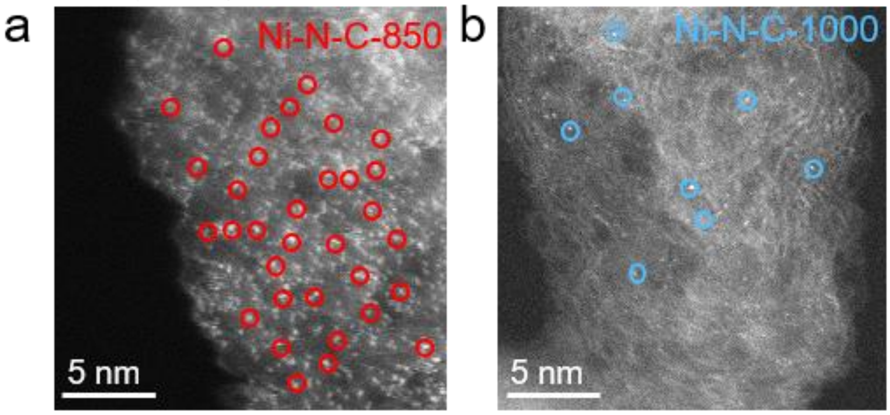

Figure S10. HAADF-TEM image of the SAC sites inside the Ni–N–C 850 (marked by the red circles) and Ni–N–C 1000 (marked by the blue circles).

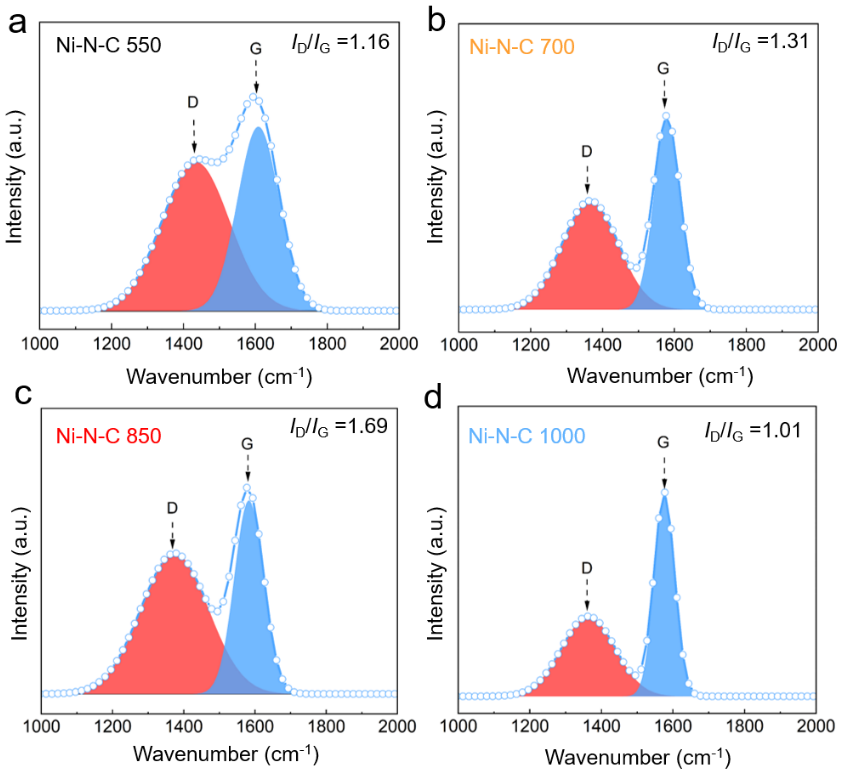

Figure S11. Raman spectra of (a) Ni–N–C 550, (b) Ni–N–C 700, (c) Ni–N–C 850 and (d) Ni–N–C 1000 samples.

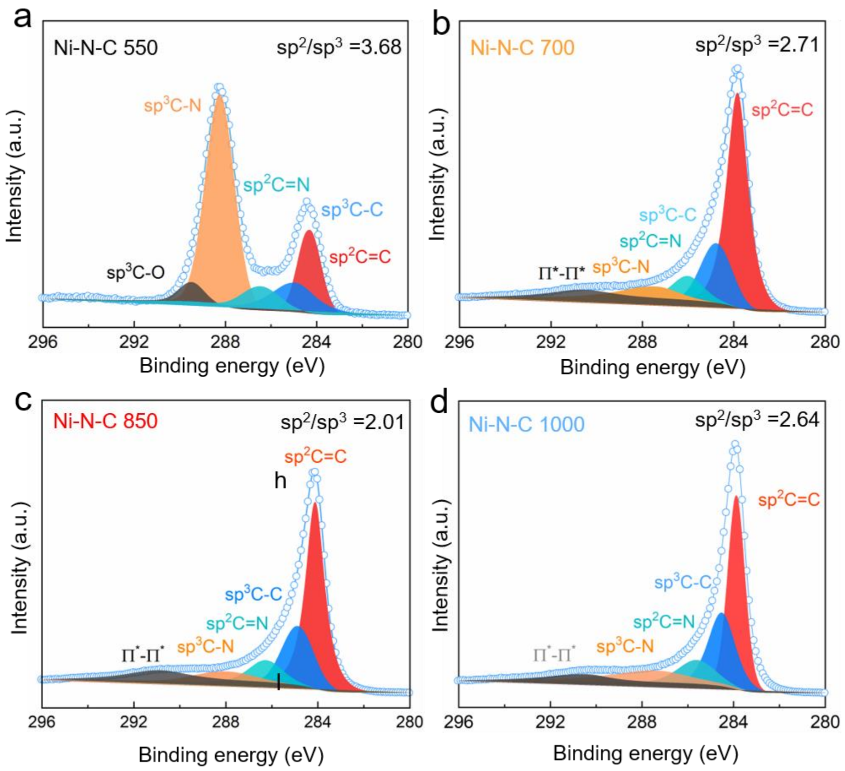

Figure S12. High-resolution C 1s XPS spectra. (a) Ni–N–C 550, (b) Ni–N–C 700, (c) Ni–N–C 850 and (d) Ni–N–C 1000 samples.

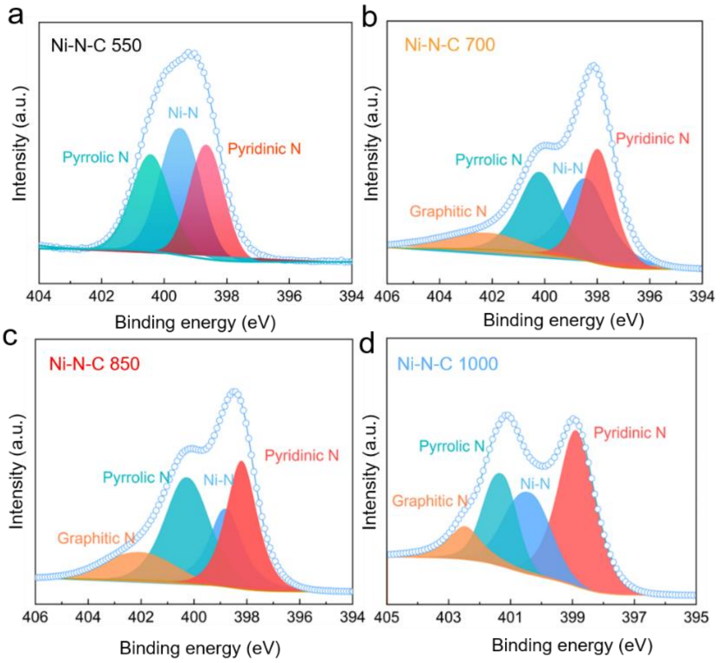

Figure S13. High-resolution N 1s XPS spectra. (a) Ni–N–C 550, (b) Ni–N–C 700, (c) Ni–N–C 850 and (d) Ni–N–C 1000 samples.

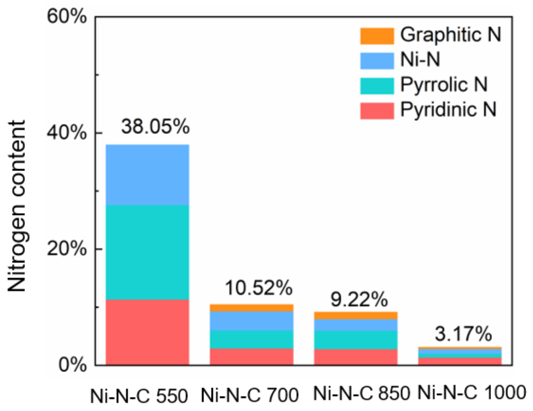

Figure S14. The statistic N contents in Ni–N–C 550, Ni–N–C 700, Ni–N–C 850 and Ni–N–C 1000 samples based on the XPS results.

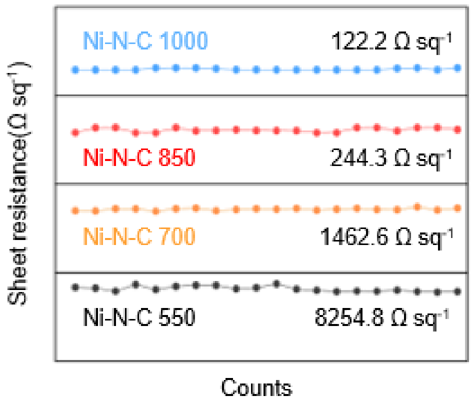

Figure S15. Sheet resistances of (a) Ni–N–C 550, (b) Ni–N–C 700, (c) Ni–N–C 850 and (d) Ni–N–C 1000 films.

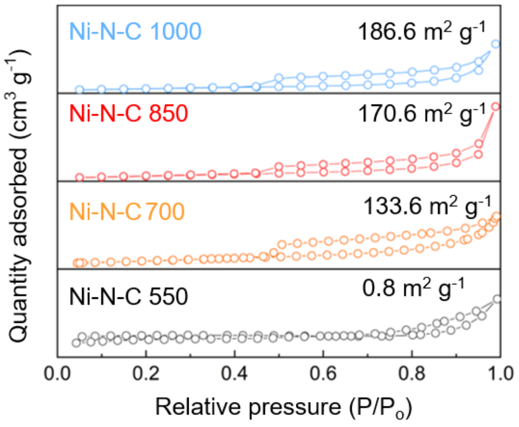

Figure S16. N_2 adsorption/desorption isotherms of Ni–N–C 550, Ni–N–C 700, Ni–N–C 850 and Ni–N–C 1000 samples.

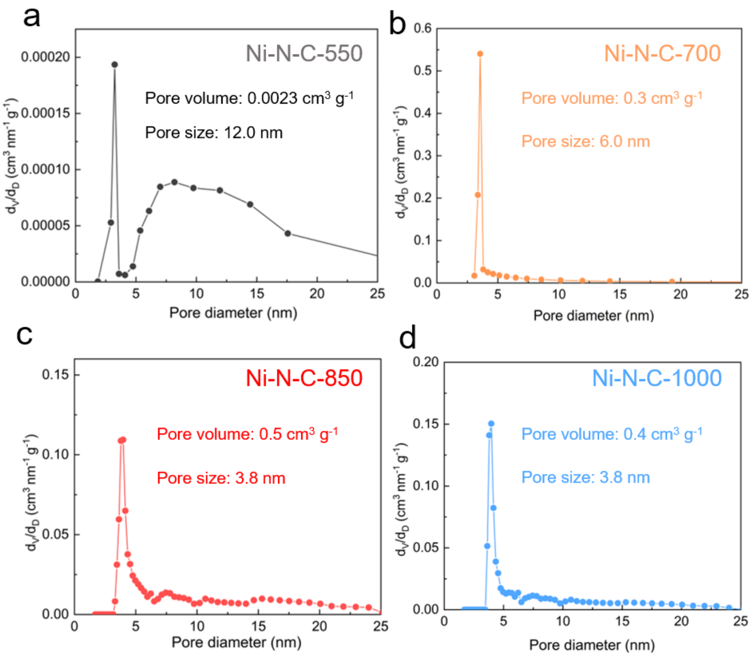

Figure S17. Pore size distributions of Ni–N–C 550, Ni–N–C 700, Ni–N–C 850 and Ni–N–C 1000 samples.

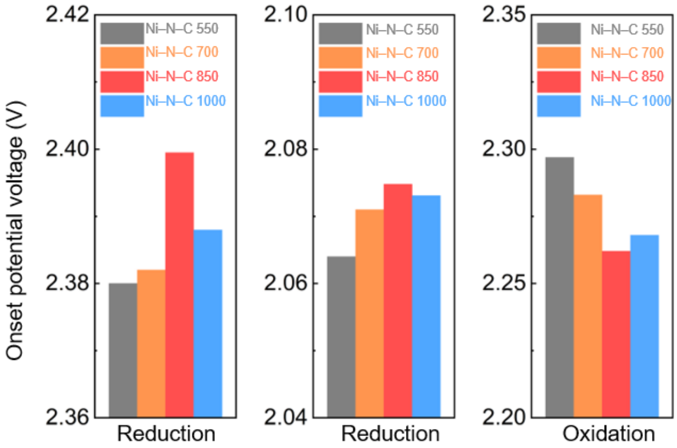

Figure S18. Related onset potentials of S/Ni–N–C cathodes in Li−S batteries extracted from the CV peaks in Figure 3n.

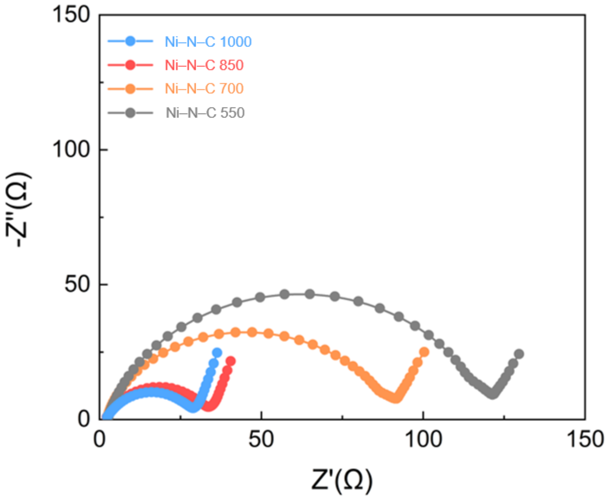

Figure S19. EIS profiles of the S/Ni–N–C cathodes.

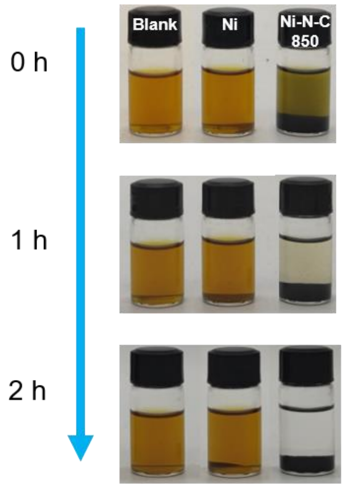

Figure S20. Visualized Li_2S_4 adsorption tests by adding Ni and Ni–N–C 850 samples.

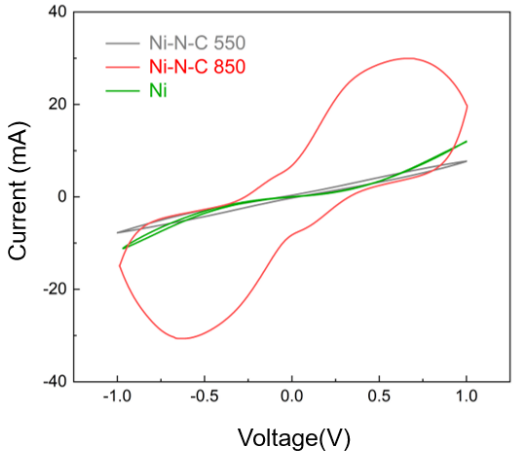

Figure S21. CV profiles of S/bare Ni and S/Ni–N–C cathodes in symmetric cells.

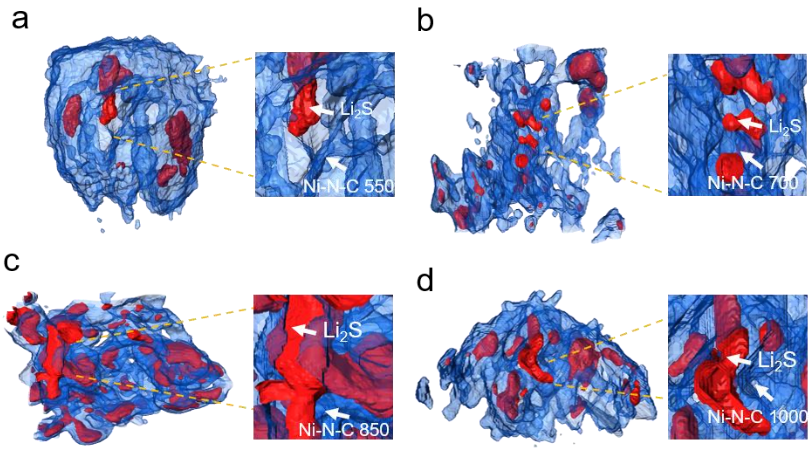

Figure S22. Synchrotron X-ray 3D nano-CT images with respect to Li_2S depositions on (a) Ni–N–C 550, (b) Ni–N–C 700, (c) Ni–N–C 850, and (d) Ni–N–C 1000 substrates.

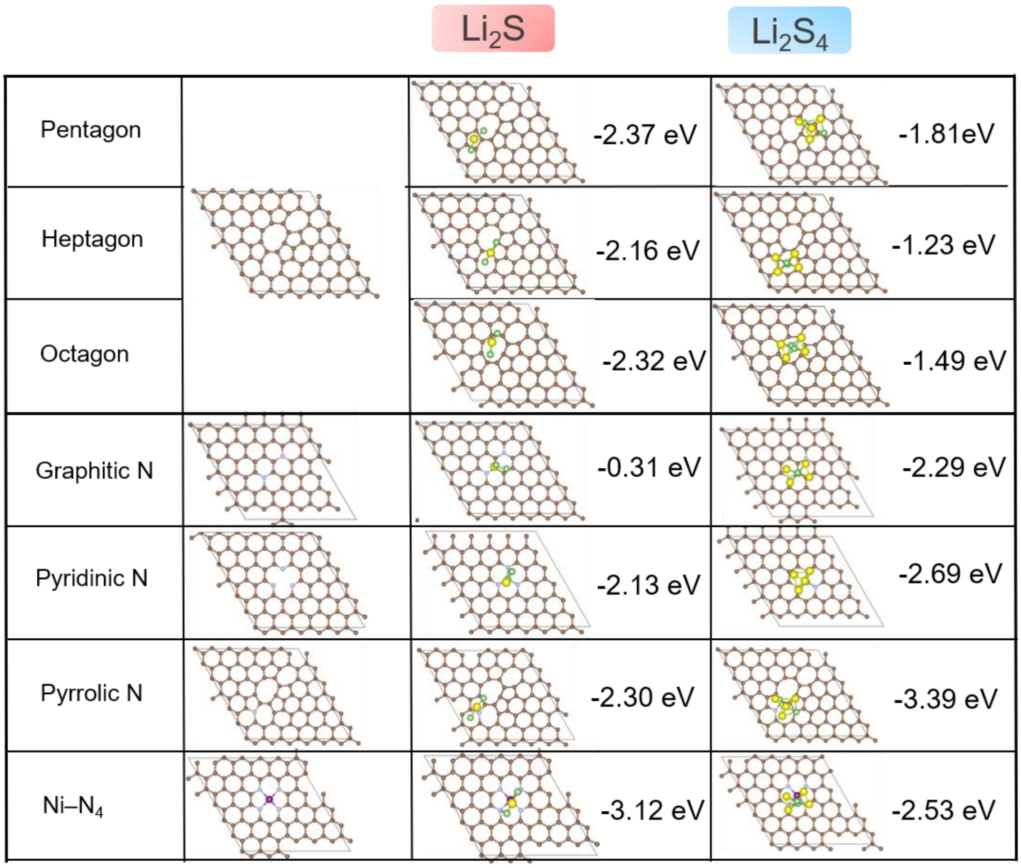

Figure S23. Adsorption configurations and the corresponding adsorption energies of Li_2S and Li_2S_4 on various active centers.

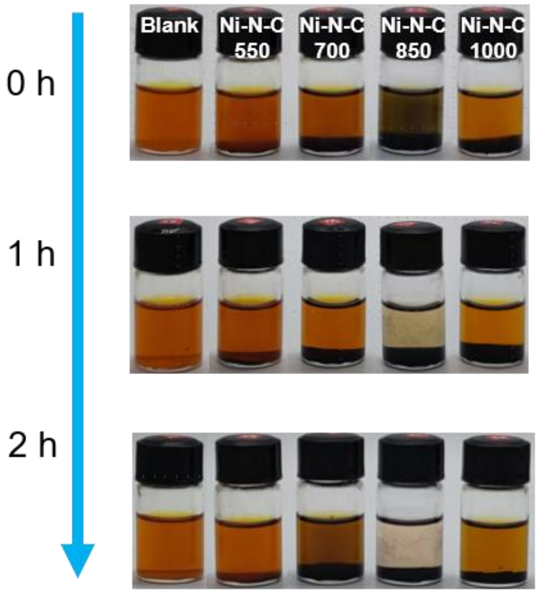

Figure S24. Visual Li_2S_4 adsorption tests by adding Ni–N–C 550, Ni–N–C 700, Ni–N–C 850, and Ni–N–C 1000 samples.

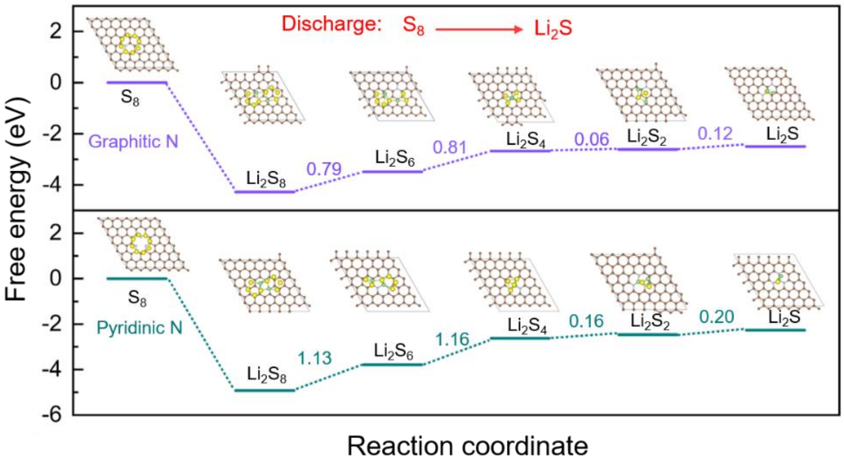

Figure S25. Gibbs free energies curves for the LiPS conversion evolution on graphitic nitrogen and pyridinic nitrogen, showing the corresponding optimized adsorption configurations.

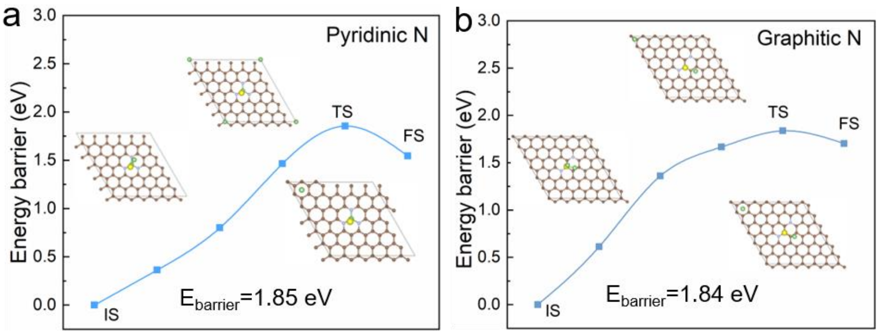

Figure S26. Dissociation energy barriers of Li_2S on (a) pyridinic nitrogen and (b) graphitic nitrogen.

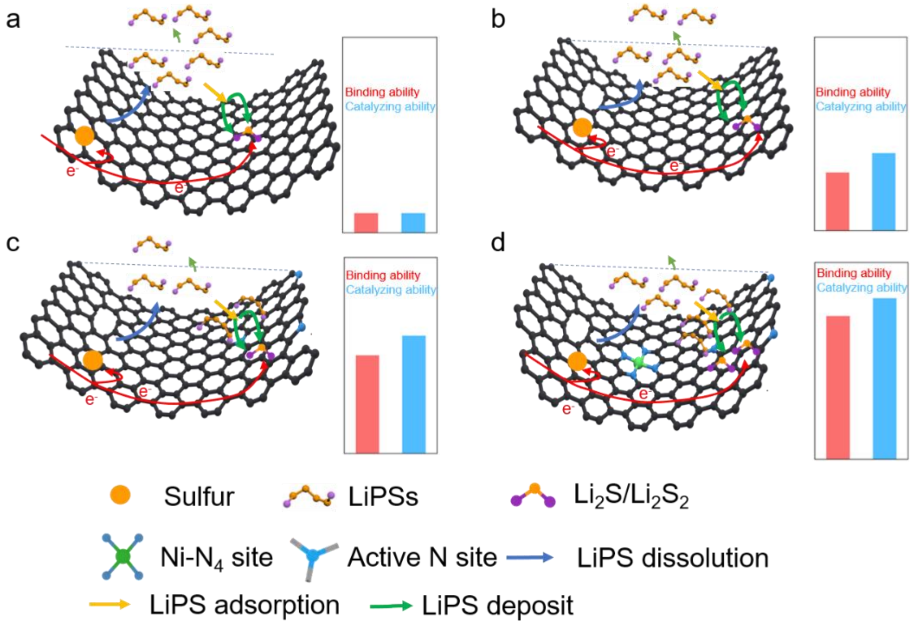

Figure S27. Scheme for the effectiveness of different active centers in immobilizing and catalyzing LiPSs. (a) Bare graphene. (b) Graphene defect site. (c) Graphene defect site + Polar N. (d) Graphene defect site +Ni–N_4+active N.

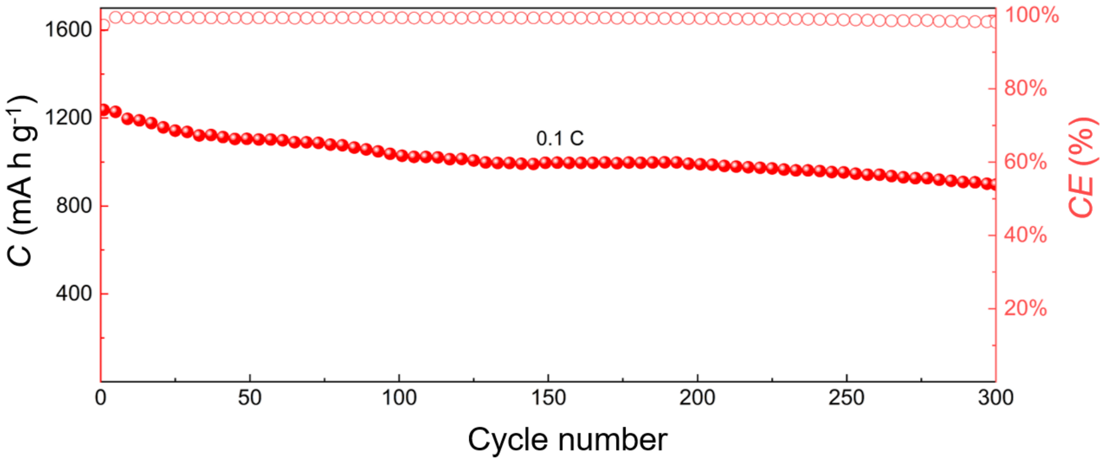

Figure S28. Cycling performance of the S/Ni–N–C 850 cathode over 300 cycles at 0.1 C.

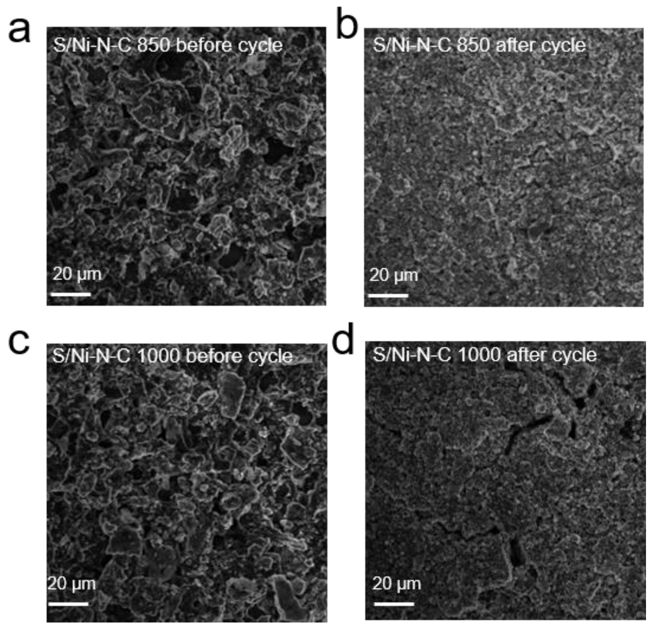

Figure S29. SEM images of S/Ni–N–C 850 cathode (a) before and (c) after 100 cycles at 0.1 C. SEM images of S/Ni–N–C 1000 cathode (b) before and (d) after 100 cycles at 0.1 C.

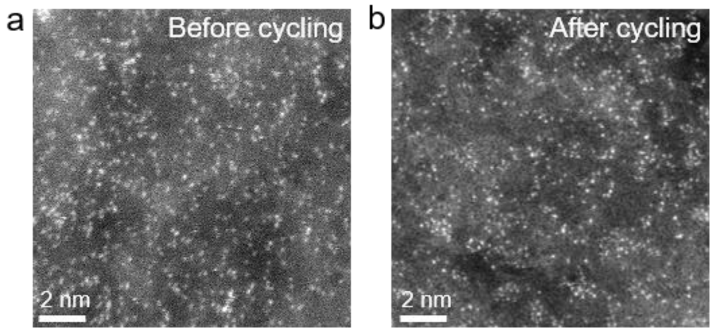

Figure S30. HAADF-TEM images of the Ni single atoms in Ni–N–C 850 before (a) and after cycling (b).

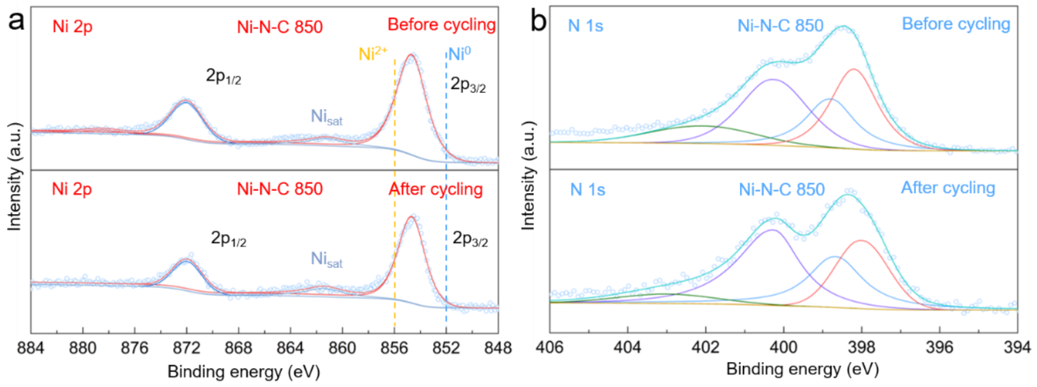

Figure S31. High-resolution XPS Ni 2p (a) and N 1s spectra (b) of Ni–N–C 850 before and after cycling.

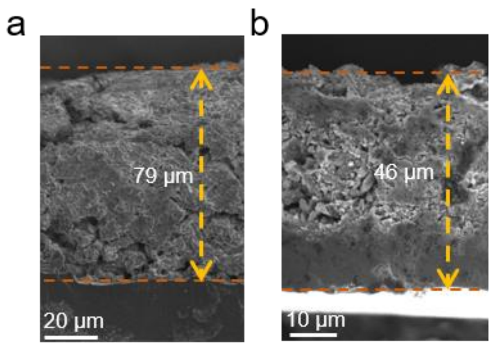

Figure S32. Cross-sectional SEM image of S/Ni–N–C cathode with the sulfur loadings of (a) 8.3 mg cm^{-2} and (b) 6.8 mg cm^{-2}.

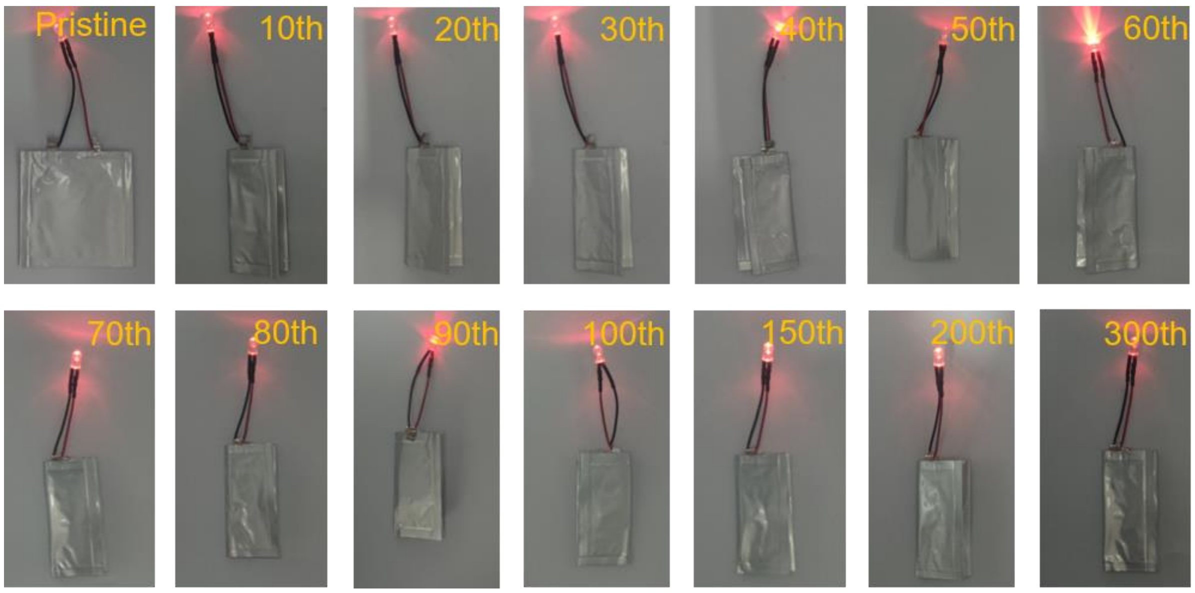

Figure S33. Digital photographs of Li–S pouch cell powering a light-emitting diode device under various folding times.

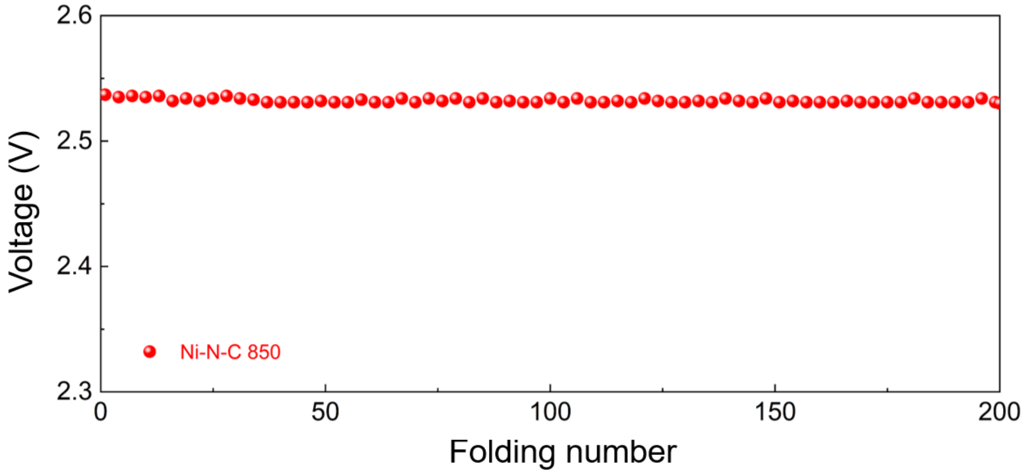

Figure S34. Folding voltage test of the S/Ni–N–C 850 cathode.

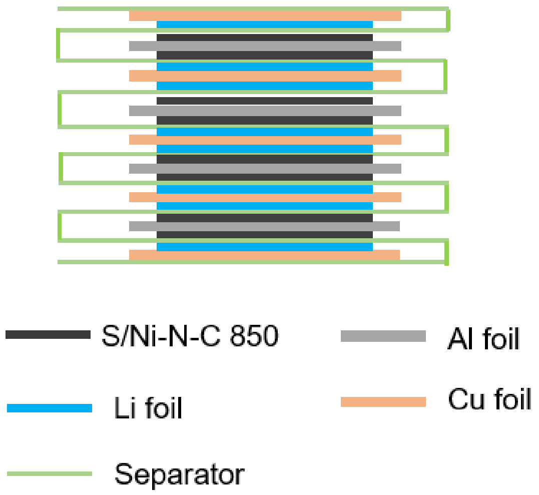

Figure S35. Configuration of interdigitated type battery winding for the pouch cell stacking.

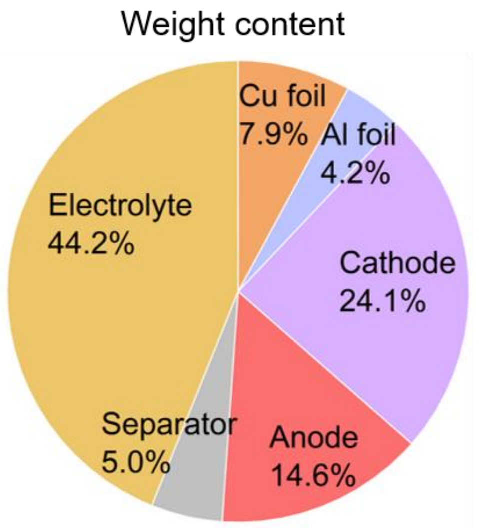

Component	Weight
Cu foil	0.19 g
Al foil	0.10 g
Cathode	0.58 g
Anode	0.35 g
Separator	0.12 g
Electrolyte	1.06 g
Total weight	2.40 g

Figure S36. Weight analysis pie chart of the 0.5-Ah-sulfur pouch cell through interdigitated type battery winding.

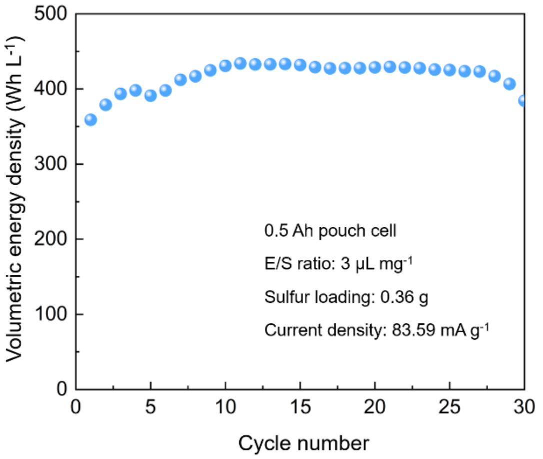

Figure S37. Volumetric energy density of 0.36-g-sulfur pouch cell with the Ni–N–C 850 as the electrocatalyst.
